# PTPRS Regulates Colorectal Cancer RAS Pathway Activity by Inactivating Erk and Preventing Its Nuclear Translocation

**DOI:** 10.1038/s41598-018-27584-x

**Published:** 2018-06-18

**Authors:** Thomas B. Davis, Mingli Yang, Michael J. Schell, Heiman Wang, Le Ma, W. Jack Pledger, Timothy J. Yeatman

**Affiliations:** 1 Gibbs Cancer Center & Research Institute, 380 Serpentine Drive, Spartanburg, SC 29303 USA; 2 Department of Biostatistics and Bioinformatics, Moffitt Cancer Center & Research Institute, 12902 Magnolia Drive, Tampa, FL 33612 USA; 3 Department of Molecular Medicine, VCOM, 350 Howard Street, Spartanburg, SC 29303 USA

## Abstract

Colorectal cancer (CRC) growth and progression is frequently driven by RAS pathway activation through upstream growth factor receptor activation or through mutational activation of *KRAS* or *BRAF*. Here we describe an additional mechanism by which the RAS pathway may be modulated in CRC. PTPRS, a receptor-type protein tyrosine phosphatase, appears to regulate RAS pathway activation through ERK. PTPRS modulates ERK phosphorylation and subsequent translocation to the nucleus. Native mutations in *PTPRS*, present in ~10% of CRC, may reduce its phosphatase activity while increasing ERK activation and downstream transcriptional signaling.

## Introduction

Colorectal Cancer (CRC) is the second leading cause of deaths from cancer in the United States^1^. Genetic analysis of CRC has recently led to a wealth of information concerning the initiation and development of CRC that may help improve our ability to treat this disease^2,3^. Activation of the RAS pathway is likely a key driver of tumorigenesis as evidenced by the fact that activating mutations of *KRAS* or *BRAF* are frequently seen in many cancers (~50% of CRCs)^3–6^. Although most CRC tumors (80%) are thought to be initiated through dysregulation of the WNT pathway, CRC tumors with activated *KRAS*/*BRAF*/*MEK*/*ERK* appear to be associated with poor outcomes^7,8^ and are difficult to treat.

EGFR inhibitors have been FDA approved for the first and second line treatment of CRC in patients with wild-type *KRAS*/*NRAS*^9–15^. Despite careful selection of wild-type RAS patients, ~60% of patients still fail to respond to these therapies, suggesting that there may be RAS pathway activation secondary to mutations in genes beyond canonical RAS pathway genes. To decipher the genes with high frequency mutations that might modulate RAS pathway activation, we carried out an integrated analysis of gene expression and sequencing data on 468 CRCs that were molecularly characterized by us recently^5,8^ using an 18-gene “RAS pathway activation” signature score^16,17^. We found that mutations in PTPRS were highly concordant with the RAS pathway signature. Moreover these mutations were identified in a significant number of cases.

PTPRS (PTPσ) is a receptor-type protein tyrosine phosphatase (PTP) whose physiological role has been well-established in the nervous system and in pituitary development, as well as in spinal cord injury and repair^18–20^. PTPRS has also been shown to play a role in ulcerative colitis, intestine epithelial permeability, autophagy regulation^21–23^, and tumor suppression^24–26^. PTPRS was recently shown to dephosphorylate EGFR in the A431 and other cancer cell lines^25–27^, and a genomic analysis revealed frequent deletion of *PTPRS* in head and neck cancers was associated with activation of the EGFR/PI3K pathway^25^. Now we report a new role for PTPRS in *negatively* regulating the RAS pathway in CRC by a mechanism modulating ERK activation. Moreover, we show that multiple, native, missense point mutations affecting various domains in ~10% of CRC patients may affect PTPRS function, underscoring their significance.

## Results

### Identification of *PTPRS* as one of the top-ranked RAS pathway signature-associated genes

**Figure 1:**
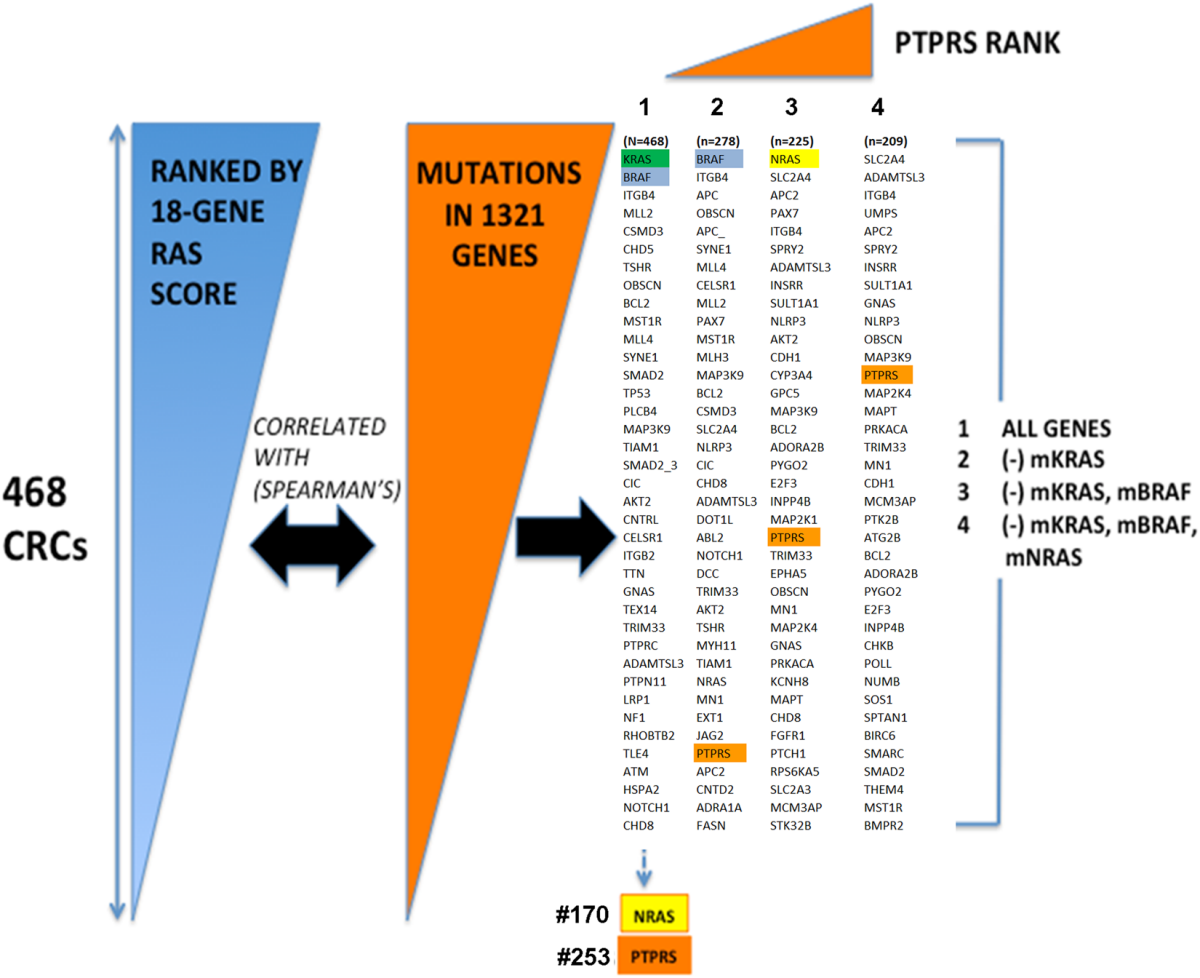
Identification of *PTPRS* by a hybrid analysis of global gene expression (Afffymetrix) and observed DNA mutations derived from targeted exome nextgen DNA sequencing of 1321 genes. 468 CRC cases were first scored for RAS pathway activity with an 18-gene RAS pathway gene expression-based activation score. *PTPRS* emerged as a lead candidate gene to activate RAS pathway when shadows of mutant *KRAS*, *BRAF* and *NRAS* were removed. See Methods for detailed description of the ranking analysis.

We recently evaluated a cohort of 468 CRC patient tumor samples using both global gene expression and targeted sequencing of 1321 cancer-related genes^5,8^. In order to identify mutated genes beyond *KRAS*, *BRAF* and *NRAS* that might account for expanded RAS pathway activity, we stratified these 468 CRCs using an 18-gene RAS pathway gene expression signature score that measures pathway activation via MEK functional output^16^. We recently adapted this signature from use in fresh frozen CRC samples to more clinically-available, archived formalin-fixed, paraffin-embedded (FFPE) tissues^17^ as a means to predict RAS pathway dependence regardless of *RAS*/*RAF* mutation status. In the ranking analysis (see Methods for detailed description) we evaluated both the correlation of mutant genes with the RAS pathway activation score and their mutational frequencies. When all patient samples (n = 468) were included, not surprisingly, the mutated gene most correlated with RAS pathway activation was *KRAS*, followed secondarily by *BRAF*. Upon removal of *KRAS*-mutated tumors (n = 278), *BRAF* became the No.1 gene (Fig. 1). When the influence of *KRAS* and *BRAF* was removed (n = 225), the ranking of *NRAS* rose from #170 to #1, and became the most correlated mutant gene, thereby validating the approach to further identify contributing mutant genes (Fig. 1). Once out of the shadow of *KRAS*, *BRAF* and *NRAS* (n = 209), a list of 15 top-ranked, potentially new RAS pathway activation-associated genes was identified, in which *ADAMTSL3*, *ITGB4*, *APC2*, *GNAS*, *PTPRS*, *ATG2B* showed >5% mutational frequency in the 209 remaining tumors, while *TGFBR2*, *SLC2A4*, *INSRR*, *NLRP3*, *MAP3K9*, *MAPT*, *MN1*, *MCM3AP* and *PTK2B* had 2.5–4.9% frequencies (see Supplementary Table 1). *PTPRS* was the most mutated, top-ranked gene (22/209, mutation frequency 10.5%), and it was also the only protein tyrosine phosphatase that stood out among sequenced phosphatases, upon removal of the masking effects of the *RAS*/*RAF* common drivers. Notably, the other 16 sequenced receptor type and non-receptor type PTPs including *PTPRT* had a much lower ranking (#223 or below). This was a surprising result given previous observations that *PTPRT* might be one of the most prominent phosphatases in CRC^28^. Interestingly, *PTPRS* was recently confirmed to be mutated in ~10% of CRC tumors in the database from the Dana Farber Cancer Center^6^. Our data show that mutations in *PTPRS* were equally present in CRC tumors with (25/257) and without (22/209) mutation-activated RAS or BRAF.

### Inhibition of PTPRS with a peptide specific inhibitor activated ERK and AKT

**Figure 2:**
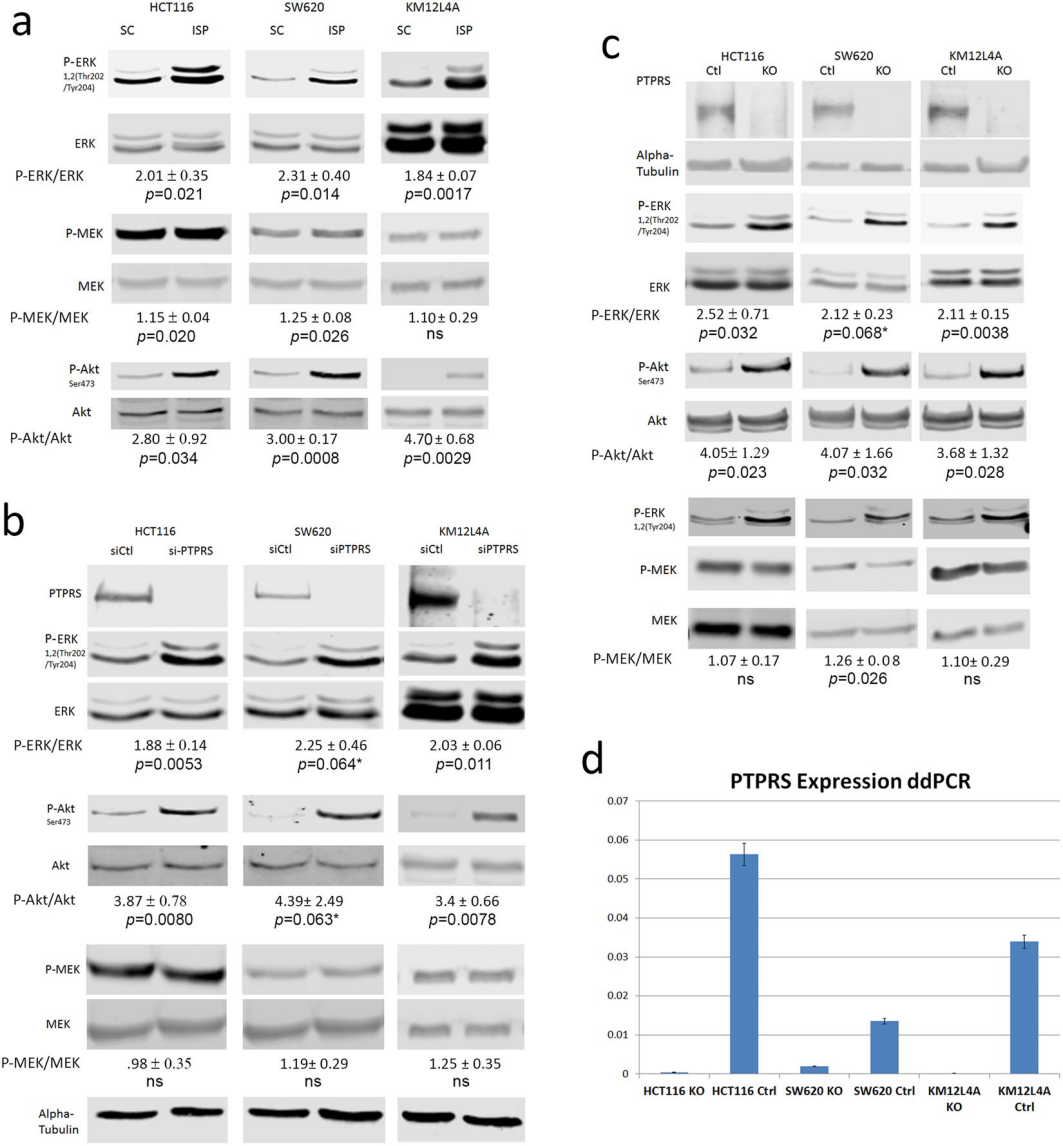
Western blot analysis for ERK and MEK activation. The indicated CRC cell lines were cultured, cells harvested, extracts prepared and western blots performed. (**a**) Cells (HCT116, SW620 and KM12L4A) cultured with ISP, an inhibitor of PTPRS, or a scrambled control peptide (SC). Western blots to detect ERK, tyrosine phosphorylated ERK, MEK and phosphorylated MEK, and AKT and phosphorylated AKT are shown as indicated. The quantitations were determined by normalizing the phosphorylated protein values with the total protein; then dividing the ISP values by the SC values. (**b**) The indicated cells had *PTPRS* knocked down with siRNA to *PTPRS* (siPTPRS) or were treated with a scrambled siRNA control (siCtl). Western blot analysis shows PTPRS, phospho-ERK, ERK, phospho-MEK, MEK, phospho-AKT, AKT, and alpha-tubulin. Knockdown of PTPRS via siRNA shows results consistent with the ISP treatments. (**c**) CRISPR knockouts of *PTPRS* in HCT116, SW620, and KM12L4A cell lines and their CRISPR control cell lines where cell extracts were used in western blot analysis for phosphorylation of ERK and MEK. This analysis shows PTPRS, ERK, phospho-ERK, MEK, phospho-MEK, AKT, phospho-AKT, and alpha tubulin in the cell line pairs (Ctl and KO) as indicated. (**d**) ddPCR analysis of *PTPRS* expression in CRISPR KO cells for HCT116, SW620, and KM12L4A. Analysis of the ddPCR result shows a near complete knockout for HCT116 and KM12L4A; SW620 shows >85% knockout. All experiments were done in triplicate. The mean and standard deviation are shown. Two-tailed, paired *t* test was used to determine the statistical significance for comparison as indicated.

To confirm a potential regulatory role of PTPRS in RAS pathway activation, we inhibited PTPRS activity *in vivo* in CRC cell lines containing both mutation-activated and wild-type *KRAS* (i.e. HCT116 (*KRAS* G13D), SW620 (*KRAS* G12V) and KM12L4A (WT *KRAS*)). All cell lines harbored wild-type *PTPRS*^29^. We employed a 33 amino acid peptide specific inhibitor of PTPRS (ISP) that has been shown to effectively inhibit PTPRS in neuronal cells^30^. Cell extracts were prepared from HCT116, SW620 and KM12L4A cells that had been treated for 24 hours with 10 μM of the ISP or a scrambled control peptide (SC). Western blot analysis was used to visualize the phosphorylation of ERK1/2, a direct indicator of RAS pathway activation. As can be seen in Fig. 2a, ISP brought about an increase in the level of ERK 1/2 phosphorylation in all three cell lines, regardless of *KRAS* activation. Notably, the ISP treatment did not bring about an increase in MEK1/2 phosphorylation in KM12L4A cells (WT *KRAS*) but caused a small (15–25%), albeit statistically significant, increase in p-MEK in HCT116 and SW620 (mutant *KRAS*) cells. We also found that vanadate, a pan tyrosine phosphatase inhibitor, also brought about an increase in ERK phosphorylation of similar magnitude in all cell lines (Supplementary Fig. 1), supporting inhibition of PTPRS phosphatase activity by ISP. In addition, we observed that ISP also increased AKT phosphorylation at S473 (Fig. 2a), suggesting that inhibition of PTPRS might mediate AKT activation as well.

### The effect of siRNA-knock down or CRISPR PTPRS knockout on activation of ERK and AKT

In order to validate the results with ISP and to confirm the specificity of action of PTPRS, we used a functionally validated *PTPRS* siRNA^24^ to selectively silence the endogenous expression of *PTPRS* in HCT116, SW620 and KM12L4A CRC cell lines. Figure 2b shows the decrease in PTPRS protein expression after siRNA treatment for 48 hours as compared to the cells treated with a scrambled siRNA. In agreement with ISP inhibition (Fig. 2a), reduced PTPRS expression in these cell lines brought about increased ERK 1/2 phosphorylation (Fig. 2b). This increase in ERK phosphorylation was verified with a second siRNA to PTPRS (Supplementary Fig. 2). In these experiments, the inhibition of PTPRS expression by siRNA did not bring about an increase in MEK phosphorylation.

To further investigate the sustained effect of the loss of PTPRS activity on ERK activation in CRC cell lines and compare to ISP inhibition and siRNA knockdown of *PTPRS*, we applied CRISPR technology to permanently knock out expression of *PTPRS* in the HCT116, SW620 and KM12L4A cell lines. *PTPRS* was successfully knocked out in each of the cell lines as seen by the loss of PTPRS protein expression (Fig. 2c) and mRNA expression (Fig. 2d). The knockout of *PTPRS* was associated with an increase in ERK 1/2 tyrosine phosphorylation (Fig. 2c). *PTPRS* KO caused a small, albeit statistically significant, increase in p-MEK in SW620 but not in HCT116 and KM12L4A cells.

**Figure 3:**
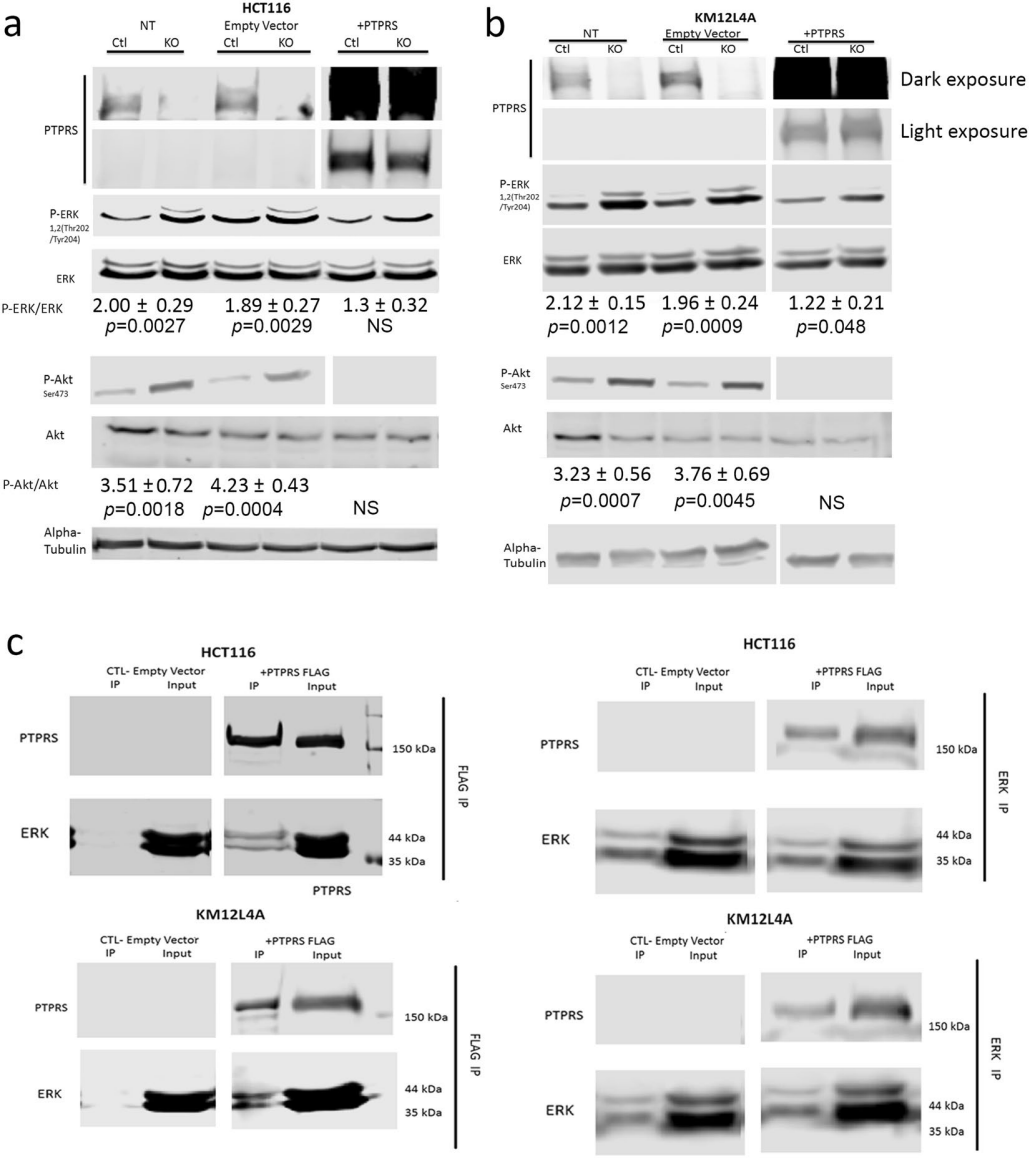
Transfections of *PTPRS* plasmid back into CRISPR KO cells decreases Phospho-ERK. HCT116 and KM12L4A CRISPR PTPRS KO cells and their CRISPR control cells were grown and transfected with full length *PTPRS* containing vector or empty vector alone. 48 hours after transfection cells were harvested and analyzed for ERK, AKT and MEK phosphorylation. Panel (a) Western blots are for HCT116 CRISPR *PTPRS* (KO) knockout cells and CRISPR controls (Ctl) as indicated. Cells not treated (NT), transfected with empty vector alone, and transfected with plasmid with *PTPRS* are shown. Panel (b) is the same as Panel (a) but with KM12L4A cells. The PTPRS expression is shown in two blots. The top blot is a darker exposure to elucidate the native PTPRS expression in the control cells. The second blot for PTPRS is a lighter exposure to show the PTPRS expression of the plasmid transfected cells. Quantitations were determined by normalizing the phosphorylated protein values with the total protein; then dividing the *PTPRS* KO by the CRISPR control values. All experiments were done in triplicate. The mean and standard deviation are shown. Two-tailed, paired t test was used to determine the statistical significance for comparison as indicated. (**c**) Co-immunoprecipitation of Flag-tagged PTPRS and ERK. Flag-tagged PTPRS was transfected into HCT116 and KM12L4A *PTPRS* KO cells. Cell lysates were then immunoprecipitated (IP) with a Flag Ab (left blots). The Flag IP was successful in pulling down the Flag-tagged PTPRS as well as pulling down ERK (lane 3 of left panel). Conversely *PTPRS* transfected cells were also immunoprecipitated using an ERK Ab (right panel). It was showed that the Flag-tagged PTPRS was pulled down along with ERK (lane 3 of right panel), again supporting a direct association between PTPRS and ERK.

To support and validate the observations seen in Fig. 2, we overexpressed PTPRS in the two CRC cell lines (HCT116 with mutant *KRAS* and KM12L4A with wild-type *KRAS*) with CRISPR knockout of *PTPRS* to determine if the exogenous expression of *PTPRS* could reduce the phosphorylation of ERK. The exogenously expressed *PTPRS* seen in Fig. 3(a,b) did reduce ERK phosphorylation that appeared independent of *KRAS* mutational activation. Thus, the data (Figs 2 and 3) indicate that PTPRS negatively regulates ERK phosphorylation.

In addition, siRNA knock-down or CRISPR KO of *PTPRS* in all the cell lines tested also increased p-AKT at S473, whereas transfection of *PTPRS* plasmid into cells completely blocked the AKT phosphorylation (Figs 2 and 3), indicating that PTPRS negatively regulates AKT activation as well.

### PTPRS co-immunoprecipitated with ERK

In order to explore if PTPRS associated with ERK, we sought to determine if immunoprecipitates of PTPRS contained ERK. HCT116 and KM12L4A cells were grown and transfected with plasmid containing *PTPRS* constructs with Flag tag or with empty vector. Cells were grown for 48 hours and then harvested for cell extract preparation. The PTPRS was immunoprecipitated with antibody to the Flag. The immunoprecipitates were analyzed by western blotting with antibodies to PTPRS-Flag and ERK. Figure 3c shows the PTPRS immunoprecipitates contained ERK and PTPRS. Conversely, when the extracts were immunoprecipitated with ERK they were shown to contain PTPRS by western blotting (Fig. 3c). These data demonstrate that PTPRS and ERK were likely associated in these cells.

### Differential effects of *PTPRS* KO in cells harboring wild-type vs. mutant *KRAS*

**Figure 4:**
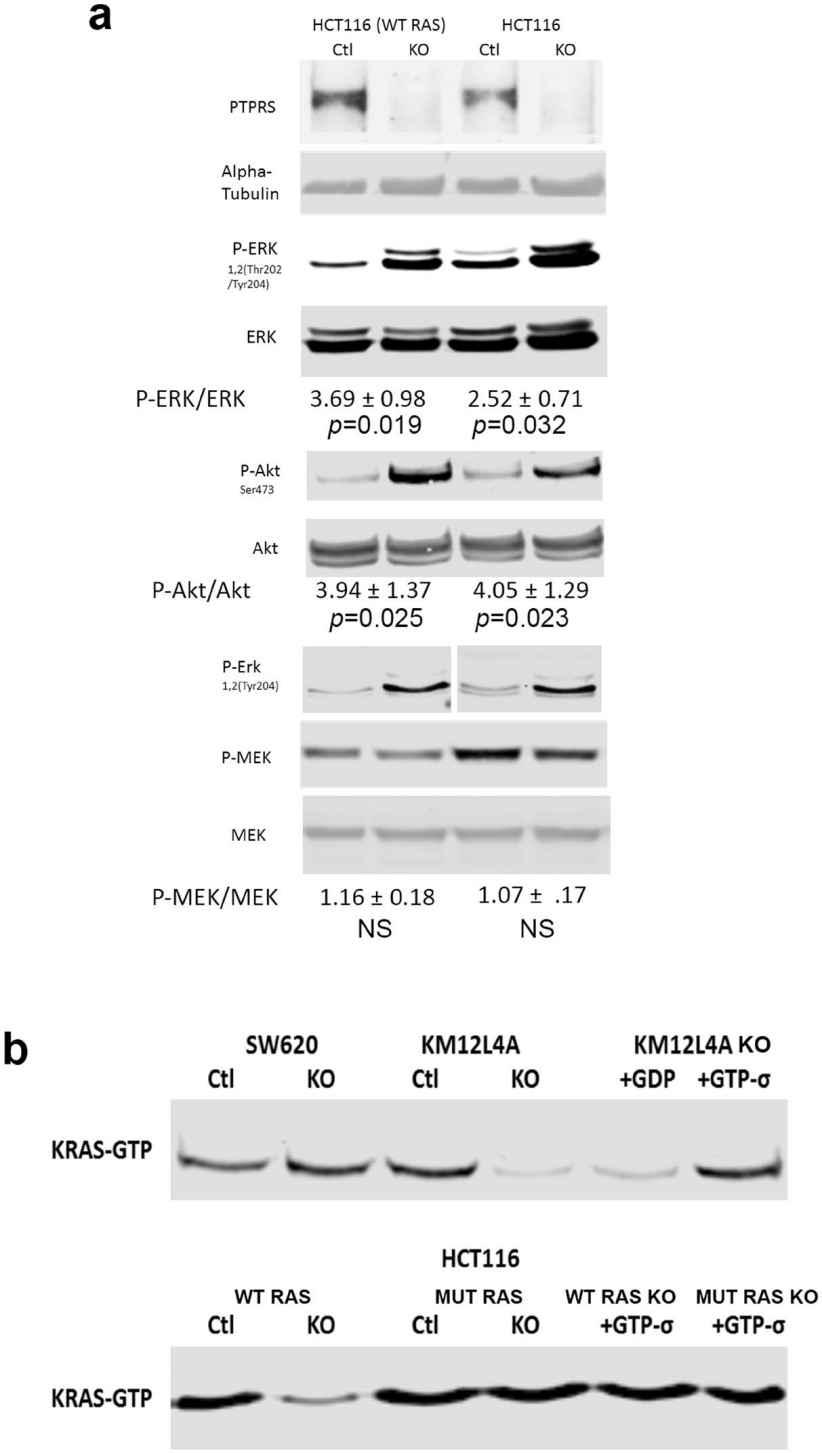
Comparison of Isogenic HCT116 Cell lines with and without activated *KRAS*. (**a**) The parental CRC HCT116 (*KRAS* G13D/+) cell line has an activating mutation of *KRAS*. Here we compare this cell line to an isogenic HCT116 (−/+) cell line that has the mutated *KRAS* allele knocked out, leaving the cell with only one WT *KRAS* allele. *PTPRS* CRISPR KO and CRISPR control paired cell lines were made in both HCT116 parental (*KRAS* G13D/+) and the isogenic HCT116 (−/+) cell lines. Extracts prepared from growing cultures were used for western blot analysis of ERK, phospho-ERK, MEK, phospho-MEK, AKT andphospho-AKT. The HCT116 (−/+) is shown to have natively less phospho-ERK compared to its parental HCT116 cell line with the activated *KRAS* mutation. The KO of *PTPRS* for both cell lines shows a dramatic increase in phospho-ERK and phospho-AKT. Quantitations were determined by normalizing the phosphorylated protein values with the total protein; then dividing the *PTPRS* KO by the CRISPR control values. All experiments were done in triplicate. The mean and standard deviation are shown. Two-tailed, paired *t* test was used to determine the statistical significance for comparison as indicated. (**b**) Active Ras pull down for all cell lines. The mutant *KRAS* cells lines, HCT116 and SW620, show a constitutively active Ras. The WT *KRAS* *PTPRS* KO cell lines, KM12L4A and the isogenic HCT116 (−/+), show a reduced amount of active Ras. KM12L4A was also used for negative and positive controls incubating the samples with GDP and GTPσ; HCT116 and HCT116 (−/+) samples were incubated with GTPσ as a positive control.

Since our data showed that loss of *PTPRS* increased the phosphorylation of ERK in tumor cells driven by both mutant KRAS as well as wild-type *KRAS*, we sought to further confirm the effects of *PTPRS* knockout (KO) on CRC cells, independent of *KRAS* mutation status. We compared the parental HCT116 cell line (*KRAS* G13D/+) with one activated *KRAS* allele to its *isogenic*, engineered derivative HCT116 (−/+) in which the activated *KRAS* allele was deleted, leaving the cell lines with only one wild-type *KRAS* allele. In each of these two cell lines, *PTPRS* was knocked out by CRISPR and paired with CRISPR controls. Cells from growing cultures of these paired cell lines were harvested. Extracts were then prepared and activated ERK was measured by western blots of phosphorylated ERK. Figure 4a shows that the cells without *PTPRS* had an increased ERK phosphorylation greater than its CRISPR control cell line. Clearly, not only did the reduction of PTPRS expression modulate the ERK activity in cells with a mutant KRAS driven ERK pathway, but an increase in ERK activation was also seen in the cells with wild-type *KRAS*. While mutant *KRAS* HCT116 (KRAS G13D/+) seemed to induce more phospho-ERK than wild-type *KRAS* HCT116 (−/+), the loss of *PTPRS* did not bring about an elevated MEK phosphorylation in either cell line.

RAS-GTP is a measure of RAS activity. It has been recently reported that activated ERK might have a negative inhibitory effect on RAS activity^31^. Interestingly, here we found that *PTPRS* KO cells show evidence of feedback inhibition of RAS-GTP expression in wild-type RAS cells (KM12L4A and HCT116 wild-type *KRAS*). This inhibition by *PTPRS* KO was not seen in mutant *KRAS* cells (SW620 and HCT116 parental) (Fig. 4b)

### The effect of *PTPRS* KO on the expression of ERK regulated genes

**Figure 5:**
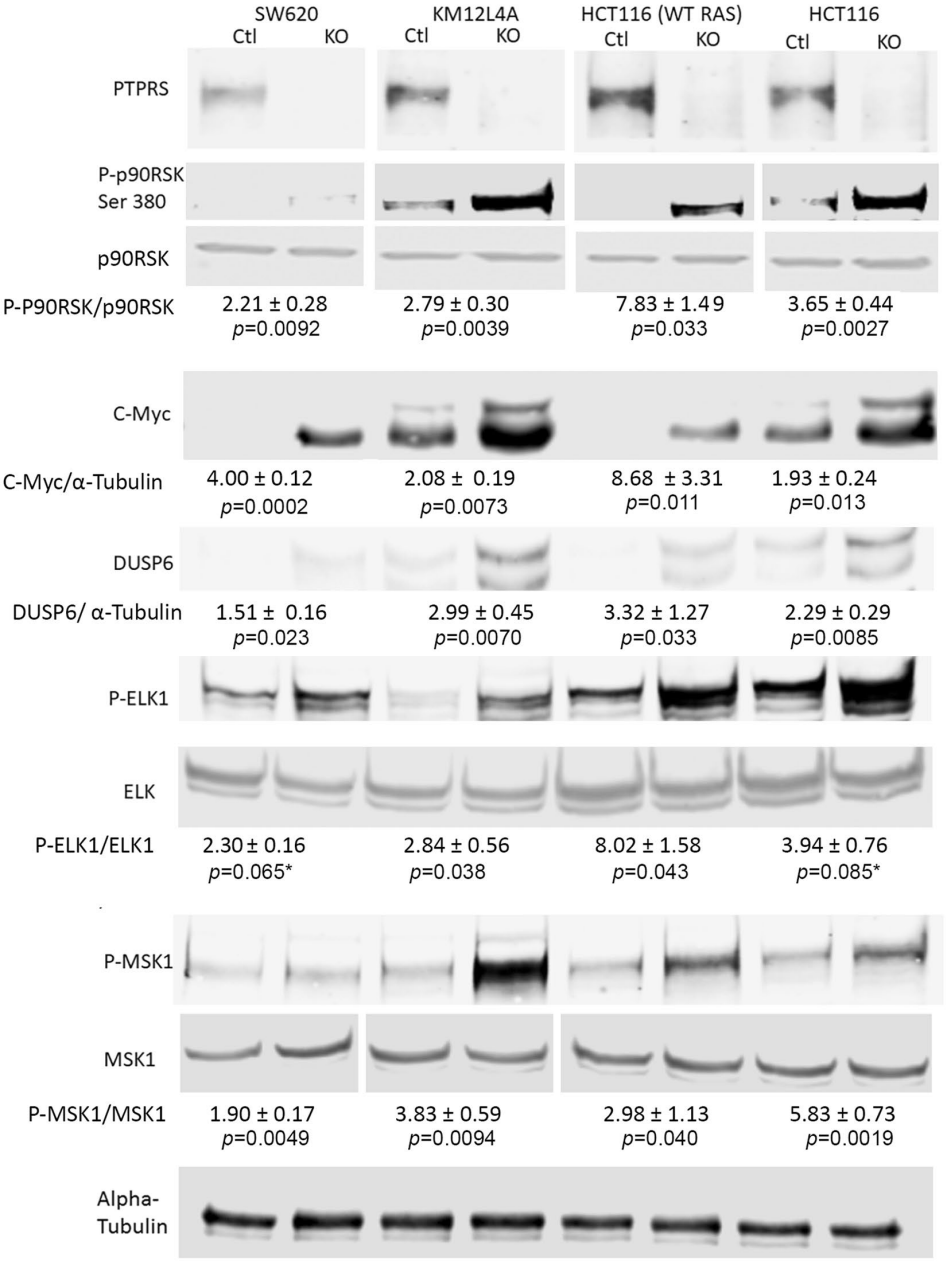
ERK targeted gene regulation and specific phosphorylations. The CRC cell lines SW620, KM12L4A, HCT116 (WT *KRAS*) [HCT116 (−/+)], and HCT116 [HCT116 (*KRAS* G13D/+)] along with their paired CRISPR* PTPRS* knockout cell lines were grown and cell extracts were prepared. Western blot analysis for Phospho-p90RSK (Ser 380), DUSP6, and C-Myc were performed. The *PTPRS* knockout cell line in each pair shows the presence of more protein for each product tested than its parental matching cell line. This effect is consistent even between the WT *KRAS* and mutant *KRAS* HCT116 cells. Phosphorylation of Elk-1 (S383) and MSK1 (T581) are shown. Quantitations were determined by normalizing the phosphorylated protein values with the total protein; then dividing the *PTPRS* KO by the CRISPR control values. The mean and standard deviation are shown.

In order to validate the effect of PTPRS on the modulation of ERK activation, we examined the protein expression of several ERK-regulated genes including c-MYC and DUSP6^32–35^. In addition, we also examined the ERK specific phosphorylation of ELK1, MSK1 and p90-RSK^33,34^. To determine and compare the effects of the loss of *PTPRS* on the expression of these genes in CRC cell lines, we compared HCT116 (*KRAS* G13D/+) to HCT116 (−/+) in paired cell lines, with and without CRISPR KO of *PTPRS*. In addition, we also investigated these same ERK regulated genes in KM12L4A (wild-type *KRAS*) and SW620 (mutant *KRAS*) *PTPRS* CRISPR KO cell lines. As seen in Fig. 5, the cells lacking *PTPRS* had a marked increase in the protein expression of ERK targeted genes and in the phosphorylation of ERK-specific downstream protein targets. Notably, HCT116 (*KRAS* G13D/+) cells that have activated *KRAS* produced higher protein expression than the HCT116 cells (−/+) with wild-type *KRAS*. The loss of PTPRS, however, brought about increased gene expression in both cell lines regardless of *KRAS* mutation status. These data demonstrate that increased ERK phosphorylation was correlated with an increase in the ERK biological response of regulated gene (protein) expression and signaling.

### The effect of *PTPRS* on EGFR signaling in CRC cell lines requires wild-type RAS

**Figure 6:**
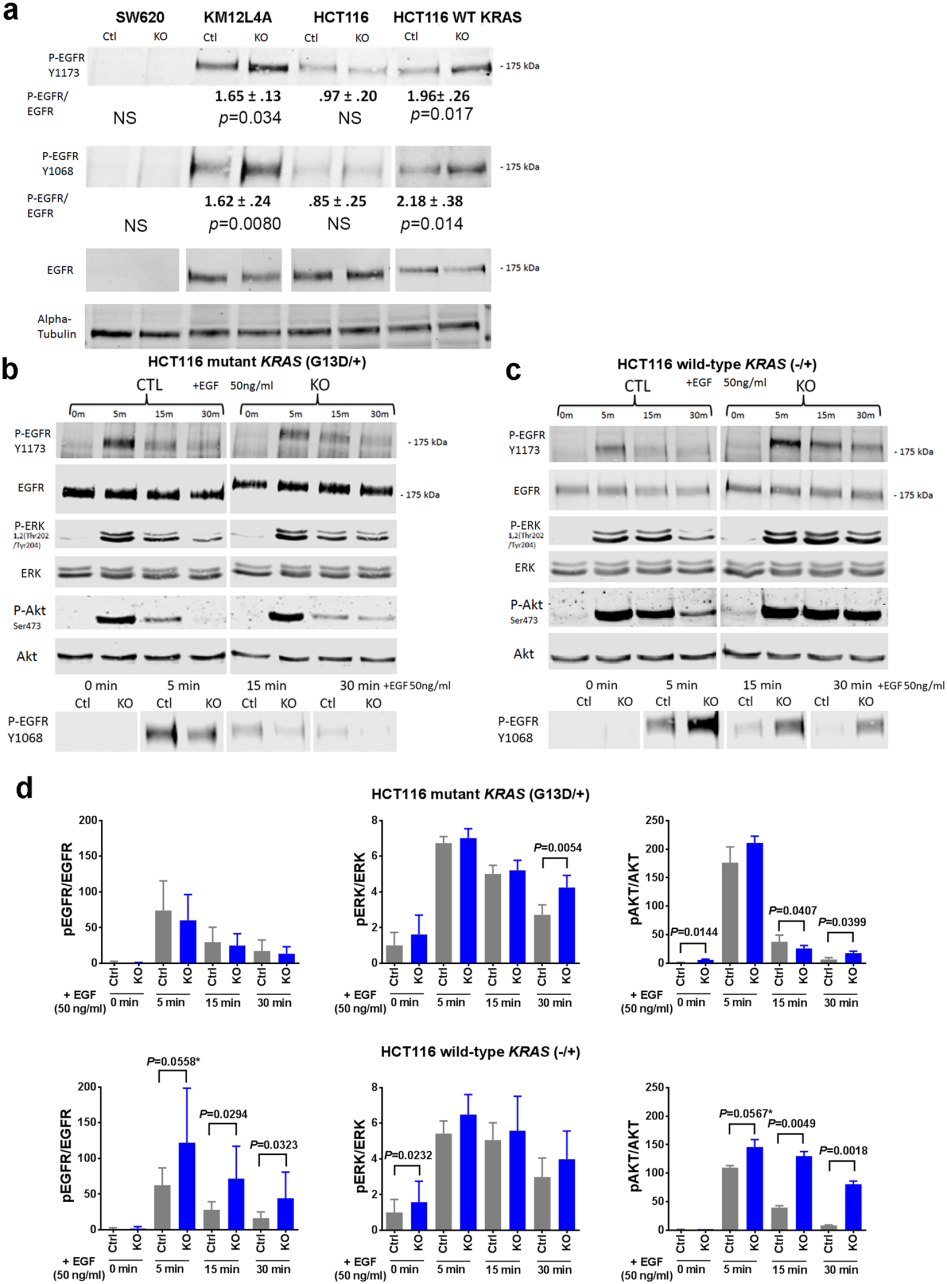
The activation of EGFR and ERK in *PTPRS* knockout cell lines and in response to EGF stimulation. (**a**) The CRC *PTPRS* CRISPR KO cell lines SW620, KM12L4A, HCT116 [mutant *KRAS* G13D/+], and HCT116 [WT *KRAS* −/+] and their CRISPR control cells were cultured, and harvested for western blotting to determine the phosphorylation of EGFR. Phosphorylation of EGFR Y1173, Y1068 and total EGFR are shown. Quantitations were done normalizing each phospho-EGFR levels against total EGFR levels; normalized phospho-EGFR values for the KO were then divided by the control values to see the fold change difference. SW620 shows no EGFR expression revealing that EGFR appears to be not the driving mechanism of the observed change in phospho-ERK. HCT116 [*KRAS* G13D/+] also shows no change in EGFR phosphorylation. The WT *KRAS* cell lines, KM12L4A and HCT116 [WT *KRAS* −/+] do show a change in phospho-EGFR. All experiments were done in triplicate. The mean and standard deviation are shown. Two-tailed, paired *t* test was used to determine the statistical significance for comparison as indicated. (**b**) Time course of serum starved HCT116 parental [mutant *KRAS*, G13D/+] *PTPRS* CRISPR KO and CRISPR control cells treated with EGF (50 ng/ml). At the times indicated, cells were harvested and extracts were prepared for western blotting. EGFR, EGFR phosphorylated at Y1173 andY1068, as well as ERK, phospho-ERK, AKT and phospho-AKT are shown. (**c**) HCT116 WT *KRAS* [−/+] *PTPRS* CRISPR KO and CRISPR control cells are shown with identical treatment as in (**b**). (**d**) This graph shows the normalized values of phospho-EGFR (left panels, average of Y1173 and Y1068), phospho-ERK (middle panels), and phospho-AKT (right panels) for the blots shown in Fig. 6b (top three panels) and Fig. 6c (bottom three panels). All experiments were done in triplicate. The mean and standard deviation are shown. Two-tailed, paired *t* test was used to determine the statistical significance for comparison. Significant *P* values (<0.05) are shown; * − near significant *P* values.

The loss of *PTPRS* brought about elevated ERK signaling in CRC cell lines as demonstrated by the increased protein expression of ERK-regulated genes. Since PTPRS was reported to be an EGFR phosphatase in other cancer cell lines^25–27^, we wanted to determine if the effect of *PTPRS* KO might be through modulating EGFR activity, upstream of RAS signaling. We first examined the level of EGFR phosphorylation at Y1068 and Y1173 in CRC cell lines, with and without expressed *PTPRS* (CRISPR KO). Figure 6a shows that no change in p-EGFR at Y1068 and Y1173 in HCT116 parental cells, whereas SW620 lacked expression of both P-EGFR and EGFR. However, we observed that *PTPRS* KO caused a modest, but statistically significant, increase in the EGFR phosphorylation at Y1068 and Y1173 in HCT116 (−/+) and KM12L4A cells both of which have WT *KRAS*. To further confirm a role of *KRAS* mutation status in *PTPRS* KO-mediated modulation of EGFR phosphorylation response, we used isogenic cell pairs HCT116 (*KRAS* G13D/+) vs HCT116 (−/+) with and without PTPRS expression. Cells were starved for 24 hours and then challenged with EGF. At the indicated times after EGF addition, cells were harvested, and the amount of phosphorylated EGFR was determined by western blotting. We found that in HCT116 (*KRAS* G13D/+) cells, *PTPRS* KO had no or minimal effect on EGFR phosphorylation (Fig. 6b,d). However, wild-type *KRAS* HCT116 (−/+) cells lacking *PTPRS* had a more prolonged activation of phospho-EGFR than cells containing *PTPRS* (Fig. 6c,d). Similar to P-EGFR, a stronger effect on the AKT phosphorylation was also seen in wild-type *KRAS* HCT116 cells (Fig. 6b–d).

### Native PTPRS mutations found in CRC decreased PTPRS activity

**Figure 7:**
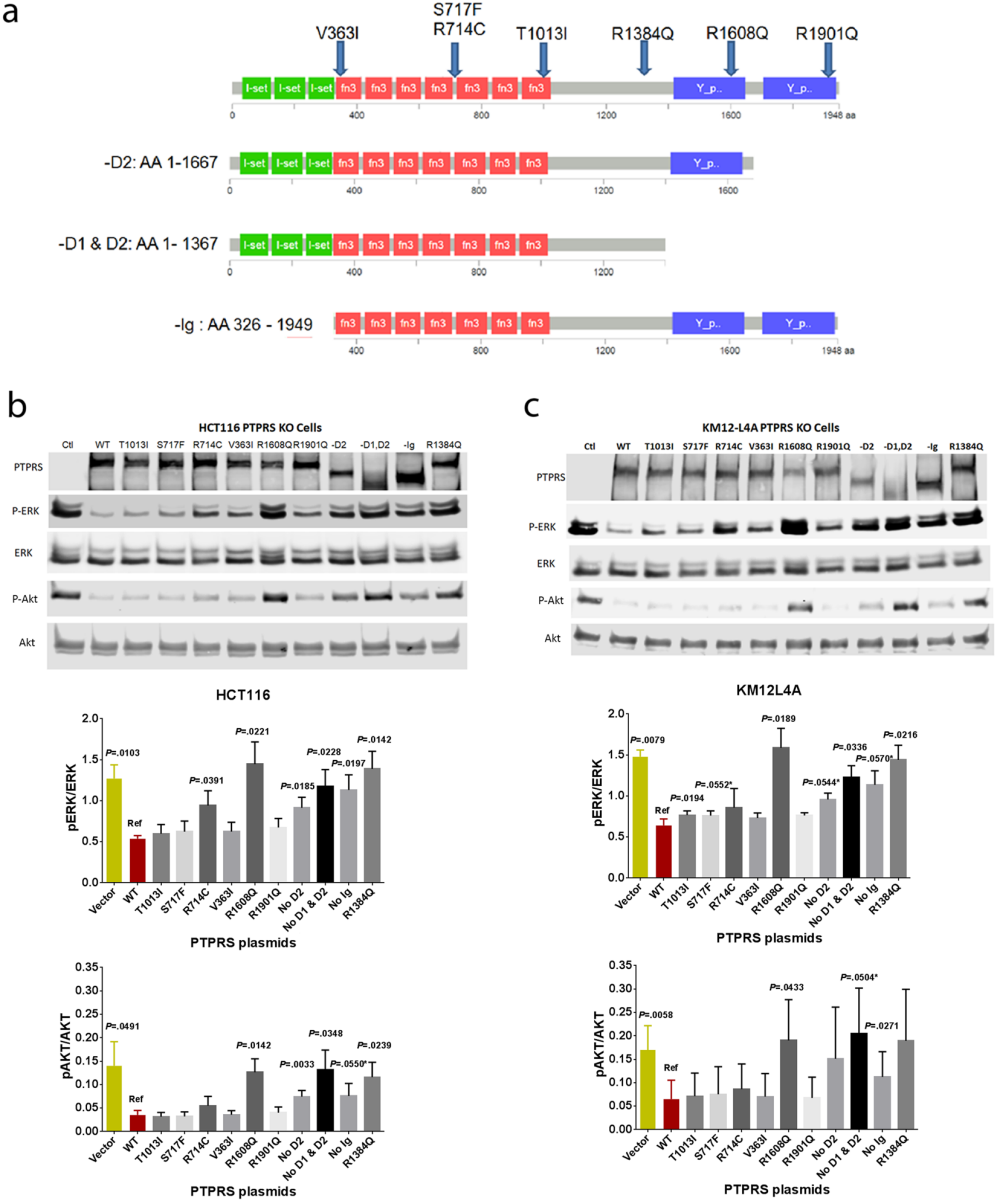
Effects of endogenous native *PTPRS* mutations on ERK de-phosphorylation assay. (**a**) Endogenous, native mutations were selected for further study based on frequency and location. 7 plasmids containing selected point mutations were synthesized. Additionally, 3 truncated mutants were synthesized. (**b**,**c**) HCT116 (*KRAS* G13D/+) and KM12L4A (WT *KRAS*) PTPRS KO cells were transfected with the various mutant plasmids and cultured for 48 hours. Extracts were prepared and levels of ERK and AKT phosphorylation were determined with western blotting. Results show that 6 of 10 mutations we tested (R714C, R1608Q, R1384Q, -D2, -D1&D2 and –Ig) exhibited completely or considerably reduced PTPRS activity compared to WT *PTPRS* plasmid. Similar results were observed in both cell lines tested. The experiments were done in triplicates. Quantitations were determined by normalizing the phosphorylated protein values with the total protein. All experiments were done in triplicate. The mean and standard deviation are shown. Two-tailed, paired *t* test was used to determine the statistical significance for comparison between WT *PTPRS* (Ref) and *PTPRS* mutants. Significant *P* values (<0.05) are shown; * − near significant *P* values; Vector − empty vector as a control.

A significant number of somatic mutations in *PTPRS* were found in our 468 tumor database, and in the Dana Farber CRC database recently published^6^. The landscape of these *PTPRS* mutations is shown in Supplementary Fig. 3. In order to determine if the mutations in *PTPRS* could alter its functionality, we performed a biochemical analysis using phospho-ERK and phospho-AKT as readouts. We selected 7 native *PTPRS* mutants in CRC for further analysis (Fig. 7a). These *PTPRS* mutants were selected based on their frequency and the position of the mutation within specific domains in the PTPRS protein structure. In addition to the native mutations, we also prepared three plasmids with deletion mutations to remove the immunoglobulin (Ig) domain or the phosphatase domains (D1 and/or D2) of PTPRS (Fig. 7a). The HCT116 (mutant *KRAS*) and KM12L4A (WT *KRAS*) CRISPR *PTPRS* KO cell lines that had highly elevated ERK and AKT phosphorylation (Fig. 2c) were used here. When full-length wild-type *PTPRS* was transfected back into the cells for 48 hours, the dramatically reduced phospho-ERK and phospho-AKT were observed compared to the empty vector control (Fig. 7b,c), indicating the inhibitory activity of PTPRS. The constructs with the appropriate *PTPRS* mutations were then transfected into HCT116 for 48 hours for western blot analysis. Results show that 6 of 10 mutations tested (R714C, R1608Q, R1384Q, -D2, -D1&D2 and –Ig) exhibited completely or considerably reduced PTPRS activity compared to wild-type *PTPRS* plasmid (Fig. 7b,c). For example, two *PTPRS* point mutations (R1608Q and R1384Q) showed a distinct reduction in ERK de-phosphorylation (i.e. phospho-ERK with the *PTPRS* mutations compared to wild-type *PTPRS* transfection). The levels of p-ERK and p-AKT seen in these mutants (lanes 7 and 12) match those of the empty vector (Control, lane 1) and the truncation mutant that removes both D1 and D2 phosphatase domains (lane 10), implying that point mutations R1608Q and R1384Q are complete, de-activating mutations. Interestingly, removal of just the D2 domain (lane 9) or of the IG domain (lane 11) appears to reduce the activity of PTPRS as measured by phospho-ERK levels, but is not completely de-activating, as is seen in lanes 7, 10, and 12. The remaining 4 mutations (T103I, S717F, V363I and R1091Q) had only minimal or modest effects on the PTPRS activity (Fig. 7b,c). Notably, similar results were also observed for phospho-AKT in HCT116 and KM12L4A cell lines.

### PTPRS expression reduced nuclear ERK staining

**Figure 8:**
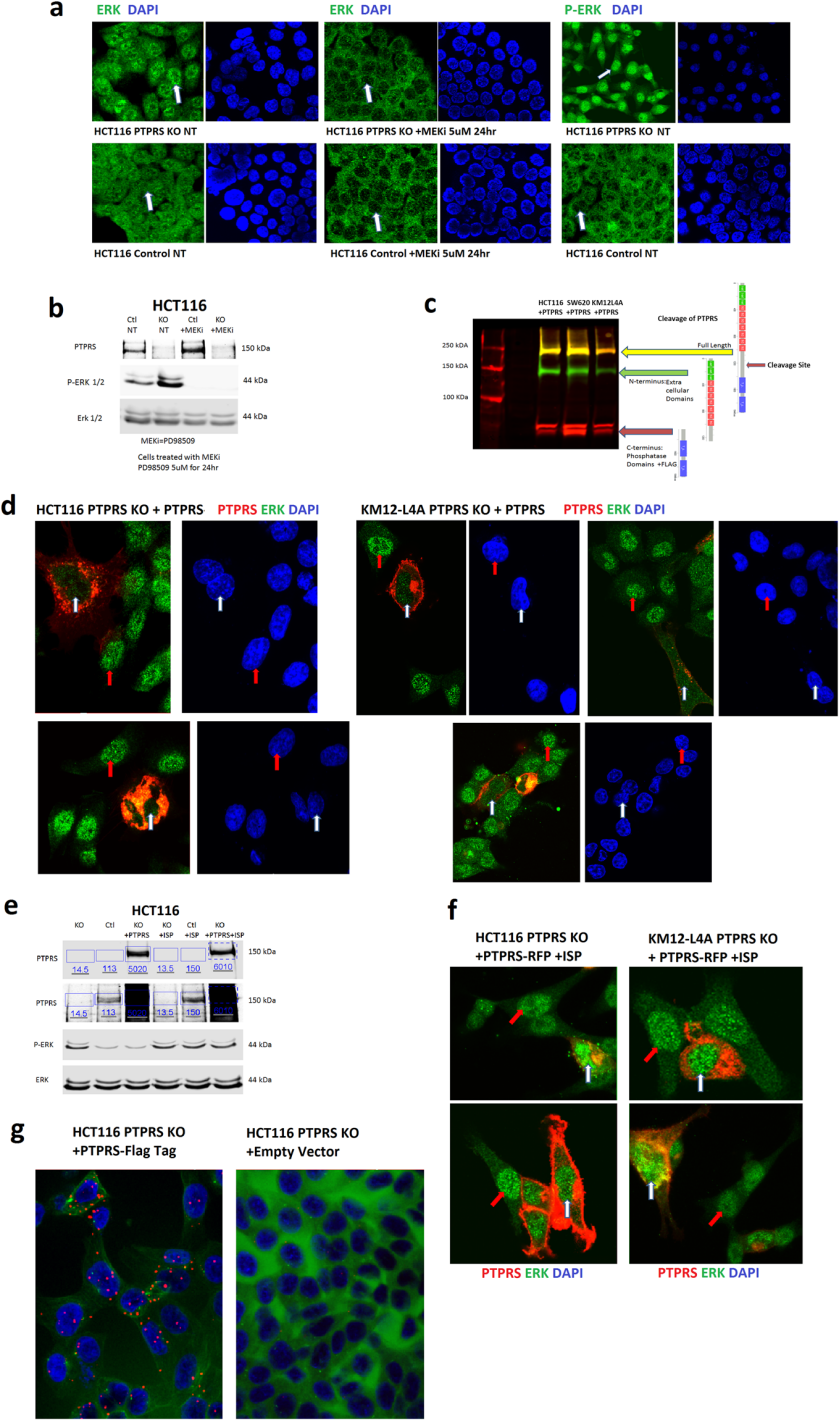
PTPRS affects the localization of ERK. (**a**) Immunofluorescent staining shows ERK nuclear localization in HCT116 PTPRS KO cells (upper left) and general whole cell staining in HCT116 control cells (lower left). A comparison of the control cells to the KO shows that the *PTPRS* KO cells have nuclei that are enriched with total ERK (arrow in top left compared to arrow in bottom left). The ERK phosphorylation in its location is confirmed with a MEK inhibitor (PD98509, middle panels). The inhibition of MEK prevent ERK phosphorylation resulting in ERK not being translocated to the nucleus (middle images have nuclei with very low-stained signals for ERK). Staining for phospho-ERK (right panels) shows a very dynamic difference with the *PTPRS* KO cells showing a bright signal and the control cells having very weak signal. Supplementary Fig. 4 shows the linear profile of fluorescence intensity for both DAPI and ERK/phospho-ERK. These measurements confirm the increased signal seen in the nuclei of *PTPRS* KO cells. (**b**) Western blot for the cells in Fig. 8a. The left two lanes are untreated HCT116 cells and right two are treated with 5 μM of MEK inhibitor PD98509 for 24 hours. The upper blot confirms the PTPRS knockout in the KO cells. The lower blots for phospho-ERK and total ERK confirm the increased phospho-ERK for the *PTPRS* KO in the left two lanes. The right most lanes confirm that the MEKi prevented the phosphorylation of ERK. (**c**) Multichannel blot of cells transfected with *PTPRS* with a C-terminal Flag tag. This blot uses both a PTPRS (rabbit green) and Flag (mouse red) antibody. Here the cleavage of PTPRS is illustrated. Full length PTPRS is 217 kDa (yellow), the N-terminal subunit containing extracellular and transmembrane domains are 140 kDa (green) the C-terminal Subunit containing phosphatase D1 and D2 domains are 78 kDa (red). This cleavage was a consistent result, and seen in all cell lines used. (**d**) Assessment of *PTPRS* transfection on ERK localization. HCT116 and KM12L4A *PTPRS* KO cells were transfected with a RFP C-terminal tagged *PTPRS*. Here we examined the localization of PTPRS (red) and total ERK (green) as well as their co-localization (orange). The *PTPRS* transfected cells (red) show a critically reduced level of ERK in their nuclei (white arrows) when compared to the cells not over expressing PTPRS (red arrows), which have bright green nuclei (ERK). (**e**) Western blotting corresponding to the cells used in 8d and 8f. The left three lanes show the *PTPRS* KO compared to the control cells and *PTPRS* KO cells transfected with *PTPRS*. The third lane shows that *PTPRS* transfected back into *PTPRS* KO cells reduces the increased phospho-ERK back to levels equivalent to the control cell line. The right three lanes show the ISP inhibited the transfected PTPRS activity allowing for increased phospho-ERK. (**f**) The nuclear reduction of ERK as a result of *PTPRS* transfection is reversed when PTPRS is inhibited by the ISP. HCT116 and KM12L4A PTPRS KO cells were transfected with the RFP tagged PTPRS and then treated with the PTPRS inhibitor ISP. The reduction in nuclear ERK (8**d**) is completely reversed (8**f**) when PTPRS is inhibited. Both the cells overexpressing PTPRS (white arrows) and non-transfected cells (red arrows) show bright ERK signal in their nuclei. Supplementary Fig. 5 shows the DAPI stains for these images. (**g**) Duo-Link *In Situ* staining for PTPRS and ERK co-localization. HCT116 *PTPRS* KO cells were transfected with a C-terminal FLAG tagged *PTPRS* or control empty vector. The cells were then labeled with a FLAG mouse Ab and an ERK rabbit Ab. The red dots indicate a successful duolink reaction, which requires both antibodies to be in close proximity. The *PTPRS* transfected cells show an ample amount of red signal (left), and the empty vector cells do not show a significant amount of signal (right). These data suggest a direct association between PTPRS and ERK.

HCT116 *PTPRS* CRISPR KO and control paired cell lines were grown and then fixed and stained for total ERK. Immunofluorescent staining (Fig. 8a) showed strong ERK nuclear localization in HCT116 *PTPRS* KO cells (upper left). By contrast, ERK staining was present throughout the cells in HCT116 control cells (lower left). A comparison of the control cells to the KO cells showed that the *PTPRS* KO cells had nuclei that were enriched with total ERK (arrow in top left compared to arrow in bottom left), which correlated with the *PTPRS* KO cells having increased levels of phospho-ERK as shown previously in western blot analysis (Fig. 2c). The importance of ERK phosphorylation with its location is confirmed when looking at ERK in these same cells and conditions with a MEK inhibitor (PD98509, middle panels). The inhibition of MEK prevented ERK phosphorylation resulting in ERK not being translocated to the nucleus (middle images have nuclei with very low-stained signals for ERK). Staining for phospho-ERK (right most columns) showed a very dynamic difference with PTPRS KO cells showing a bright signal and control cells displaying very weak signal. The linear profile of fluorescence intensity for both DAPI and ERK/phospho-ERK confirmed the increased signal seen in the nuclei of *PTPRS* KO cells (Supplementary Fig. 4). The effect of the MEK inhibitor was also verified by Western blotting (Fig. 8b). Figure 8c shows multichannel blotting of cells transfected with PTPRS with a C-terminal Flag tag to illustrate the natural cleavage of PTPRS^36^. Full length PTPRS is 217 kDa (yellow), the N-terminal subunit containing extracellular and transmembrane domains are 140 kDa (green - PTPRS antibody) the C-terminal subunit containing phosphatase D1 and D2 domains are 78 kDa (red - Flag antibody). Notably, this cleavage was a consistent result, as seen in all cell lines used.

An effect of *PTPRS* transfection on ERK localization was further assessed. HCT116 and KM12L4A *PTPRS* KO cells were transfected with a RFP C-terminal tagged PTPRS (Fig. 8d). For PTPRS, we see two localizations: (1) the cell membrane and (2) surrounding the nucleus. The perinuclear PTPRS is likely the cleaved form of the protein that includes the C-terminal D1 and D2 phosphatase domains. The *PTPRS* transfected cells (red) showed a critically-reduced level of ERK in their nuclei (white arrows) when compared to the cells not over expressing PTPRS (red arrows), which have bright green nuclei (ERK). In addition, the nuclear reduction of ERK as a result of *PTPRS* transfection was verified by addition of the PTPRS inhibitor ISP and Western blotting (Fig. 8e,f).

We have previously shown that PTPRS and ERK co-immunoprecipitated with each other (Fig. 3c). Moreover, we used DuoLink (22,23) to further confirm that ERK and PTPRS are proximally-associated in cells (Fig. 8g). These data suggest a direct association between PTPRS and ERK.

## Discussion

Receptor type and non-receptor type protein tyrosine phosphatases (PTPs) are thought to be important in regulating the RAS/ERK pathway, although their functional role in cancer is much less understood than their counterpart protein tyrosine kinases (PTKs)^37–39^. For example, receptor-type PTPs PTPRE (PTPε) and PTPRJ (DEP-1) were shown to inhibit ERK activation *in vitro* using NIH3T3, HEK293 and/or HeLa model cell lines^40,41^. Recently, PTPN11 (SHP2), a non-receptor type PTP, was reported to play an oncogene-like role in laryngeal cancer, hepatocellular carcinoma and glioblastoma, and the mechanism appeared to involve dephosphorylating RAS to activate the RAS/ERK pathway^42–44^. Moreover, while genetic and epigenetic alterations in a number of PTPs have been observed in CRC^3,5,6,28,38,45,46^, a role for these PTPs in regulating RAS/ERK pathway is not yet known.

We identified *PTPRS* mutations as significantly associated with RAS pathway activation (Fig. 1), suggesting a regulatory role for PTPRS in RAS/ERK signaling in CRC. *PTPRS* was frequently mutated in our CRC dataset (46/468, 9.8%). This is in close agreement with the somatic mutation rate reported for *PTPRS* by DFCI (57/619, 9.2%)^6^ (Supplementary Fig. 3). Since PTPRS was reported to have a tumor suppressor-like role^24–26^, we postulated that the somatic mutations of *PTPRS*, if functional, might be *inactivating* mutations, which could mediate RAS/ERK pathway activation, which is a driver of tumorigenesis^3–6^. In support of this notion, our biochemical analyses using a specific peptide inhibitor, siRNA and CRISPR knockout demonstrated that inhibition or loss of PTPRS resulted in elevated ERK phosphorylation in both mutant *KRAS* and wild-type *KRAS* CRC cell lines (Fig. 2). The increase in ERK phosphorylation was associated with an increase in ERK-stimulated gene expression (DUSP6, CMYC) and ERK-specific phosphorylation of p90RSK, ELK1 and MSK1^32–35^ (Fig. 5). The role of PTPRS in regulating ERK activation was also confirmed by using a *PTPRS* expression plasmid, which reduced ERK phosphorylation (Fig. 3), indicating that PTPRS is a negative regulator of ERK activation.

PTPRS was reported to be an EGFR phosphatase in A431 epidermoid carcinoma cells and head and neck cancers^25,27^. We observed that loss of *PTPRS* (knockout) caused a modest but statistically significant increase in phospho-EGFR at Y1068 and Y1173 in wild-type *KRAS* CRC cell lines yet had no effect in mutant *KRAS* cell lines (Fig. 6a). Using HCT116 parental (*KRAS* G13D/+) and the *isogenic* HCT116 (*KRAS* −/+) cell line, we demonstrated that loss of *PTPRS* had no or minimal effect on EGFR phosphorylation in mutant *KRAS* HCT116 cells following EGF stimulation whereas wild-type *KRAS* HCT116 (−/+) cells lacking *PTPRS* had a more prolonged activation of phospho-EGFR compared to the control cells containing *PTPRS* (Fig. 6c,d). These data indicate that PTPRS may be involved in negative regulation of EGFR signaling in the absence of oncogenic activation of *KRAS* in CRC. Since we consistently observed a significant increase in ERK phosphorylation by inhibition/loss of PTPRS in both mutant and WT *KRAS* cell lines, the regulation of EGFR signaling appears to be not necessary for PTPRS’s role in moderating ERK activation. Activated ERK not only mediates RAS pathway downstream signaling that regulates various cellular process but can also mediate feedback regulation of RAS pathway^32–35,47,48^. We also observed *PTPRS* KO-mediated feedback inhibition of RAS-GTP expression in association with ERK activation in WT *KRAS* but not mutant *KRAS* cell lines (Fig. 4b). This suggests that mutation-activated RAS might block feedback regulation of RAS pathway activation by PTPRS.

MEK is the only known ERK kinase^34,35,47,48^. Except for a slight increase in p-MEK in SW620 cell line, the inhibition/loss of PTPRS in all other cell lines (regardless of RAS mutation status) did not alter MEK phosphorylation (Figs 2 and 4). This suggests that ERK activation observed in all cell lines tested was not mediated by MEK. We found that PTPRS and ERK co-immunoprecipitated and co-localized (Figs 3c and 8g), suggesting a direct interaction between PTPRS and ERK. Using a p-ERK Y204-specific antibody, we observed significantly-increased tyrosine-specific phosphorylation in ERK1/2 induced by *PTPRS* KO in all cell lines (Figs 2c and 4a).

Activation of ERK is required for its entry into the nucleus and its nuclear activities^47,49,50^. We found that the loss of PTPRS results in enriched nuclear localization of ERK, whereas the ectopic expression of PTPRS retains ERK primarily in the cytoplasm (Fig. 8). Thus, PTPRS may inhibit ERK nuclear localization by negative regulation of ERK activation. Alternatively, it is also possible that PTPRS may directly associate with ERK in the cytoplasm by a mechanism involving scaffold protein complexes that are thought to block ERK translocation to the nucleus^47^. PTPRS is a membrane receptor PTP that was reported to be proteolytically cleaved into two subunits (the E subunit containing the N-terminal extracellular domain and the P subunit containing the C-terminal phosphatase domains^36^. In the western blot analysis, we saw both subunits as well as the full-length protein (Fig. 8c). Thus, PTPRS might be associated with ERK on the plasma membrane. Whether PTPRS might be associated with the ERK P-subunit in the cytosol is not yet clear.

Deletion of PTPRS was reported to be associated with abnormal activation of PI3K/AKT signaling in head and neck cancers^25^. We also found that PTPRS inhibition/KO in CRC cell lines, regardless of RAS mutation status consistently increased AKT phosphorylation at S473, indicating activation of AKT (Figs 2 and 4). There exists a crosstalk between RAS signaling and PI3K/AKT signaling^47,51–53^. However, whether increased phosphorylation of ERK and AKT by *PTPRS* KO were inter-dependent is not known yet. MEK has been suggested as a focal point for cross-cascade regulation^51^. However, inhibition/loss of *PTPRS* Increased p-ERK and p-AKT without moderating p-MEK in most of cell lines tested. Thus, MEK likely did not play a role here.

Finally, it was important to assess the functional effects of a variety of the observed, native *PTPRS* mutations. We sought to test the most commonly observed somatic alterations (Fig. 7). Surprisingly, when compared to wild-type *PTPRS*, 4/7 tested native variants had a measurable deleterious effect on PTPRS function as measured by ERK activation (Fig. 7). Moreover, we were able to demonstrate a functional role for the Ig-SET domain and for the D1 + D2 domains via deletion constructs. These data suggest that a substantial percentage of the ~10% SNVs observed in CRC may have a deleterious functional effect.

RAS and its downstream effectors have been targeted to develop therapeutic inhibitors in various cancers with frequently mutated *KRAS* or *BRAF*^54–56^. Although efforts to directly target RAS have not been successful to date, selective BRAF and/or MEK inhibitors have shown clinical efficacy in *BRAF*-mutant melanoma^56^. More recently, specific inhibitors of ERK have been also developed, which appear to hold promise to overcome acquired resistance to MEK inhibitors^56–60^. It is noteworthy that the combination of MEKi with the PI3K/AKT/mTOR inhibitors has been conducted in preclinical studies and in clinical trials^61^. Our study revealed that inactivation of *PTPRS* enhanced the activation of ERK and AKT, which may facilitate the development of effective targeted therapies against ERK and/or AKT in colorectal cancer.

## Methods

### Cell Culture

HCT116 (*KRAS* G13D), SW620 (*KRAS* G12V) and KM12L4A (WT *KRAS*) CRC cell lines were obtained from ATCC and tested monthly for mycoplasma contamination with Sigma LookOut® Mycoplasma qPCR Detection Kit (Cat No. MP0040A-1KT). The parental HCT116 CRC cell line (*KRAS* G13D/+) and the engineered HCT116-*KRAS* (−/+) cell line, were obtained from Horizon Discovery (Cat.No.HD104-008). Cells were cultured in RPMI 1640 (Gibco) supplemented with 10% FBS and 1% penicillin and streptomycin.

### Immunoblotting and Active Ras Assay

Cells were lysed in 1x RIPA buffer (9806 Cell Signaling) containing 10 mM PMSF, Protease Inhibitor Cocktail (M250 Amresco), Phosphatase Inhibitor Cocktail 2 (P5726 Milipore), and Phosphatase Inhibitor Cocktail 3 (P0044 Milipore) followed by immunoblotting using LI-COR Odyssey® CLx Imaging System. Antibodies were typically duplexed using rabbit antibodies for phosphorylated antibodies and mouse antibodies for total protein. Li-Cor secondary antibodies, Goat anti-Rabbit IRDye 680RD and Goat anti-Mouse IRDye 800CW, were used with the duplexed primary antibodies.

All primary antibodies were rabbit unless specified and were sourced as follows: PTPRS (mouse Cat.No.ab55640 Abcam); PTPRS (goat AF3430 R&D Systems); alpha-Tubulin (mouse sc-8035 Santa Cruz). All other antibodies were obtained from Cell Signaling: phospho-Erk1/2 T202/Y204 (Cat.No.4370); phospho-Erk1Y204/Erk2 Y187 (mouse D1H6G); Erk1/2 (mouse 4696); phospho-MEK1/2 S217/221 (9154); MEK (mouse 4694); phospho-EGFR Y1173 (4407); EGFR (mouse 2239); Elk-1(rabbit Ab 9182), p-Elk1 (Ser383 mouse Ab 9186), MSK1 (rabbit Ab 3489), p-MSK1 (Thr 581 rabbit Ab 9595) and Erk Rabbit Ab 4695).

Active Ras assay was performed using the Active Ras Pull Down and Detection kit from Thermo Fisher (Cat.No.16117).

### RT-PCR and ddPCR

Total RNA was isolated using Autrum Total RNA Mini Kit (Cat.No.7326820 Bio Rad) followed by reverse transcription reactions with SuperScript III First-Strand Synthesis System (18080051 Thermo Fisher). ddPCR was performed using the QX200 droplet generator and reader system (Bio Rad) with ddPCR Supermix for Probes (186-3026 Bio Rad).

Samples were run in triplicate using 20 μL of final reaction mix with probes and 30 ng of cDNA per reaction. They were thermocycled on a C100 Touch Thermo Cycler using the recommended program cycle. Individual FAM probes were obtained from Bio Rad unless specified otherwise: PTPRS (dHsaCPE5055124) and the reference gene B2M HEX Probe (dHsaCPE5053101).

### Intracellular Sigma Peptide (ISP)

The Intracellular Sigma Peptide (ISP) for inhibiting PTPRS activities and the scrambled ISP were designed and reported (19). These two peptides were synthesized by GenScript at >75% purity. Both peptide sequences contain TAT domain to enable membrane penetration. (1) H-ISP (GRKKRRQRRRCDMAEHTERLKANDSLKLSQEYESI) – targeted specifically against a highly conserved 24-amino-acid intracellular wedge domain of human PTPRS and (2) Scrambled ISP (GRKKRRQRRRCIREDDSLMLYALAQEKKESNMHES) – the sequence except TAT domain is scrambled. 10 μM of ISP (the scrambled ISP as a control) was used to inhibit PTPRS in CRC cell lines.

### siRNA Transfection

Two *PTPRS*-specific siRNAs were obtained from Qiagen: PTPRS_5 siRNA (SI02759288 Qiagen, target sequence: CAGGACATTCTCTCTGCACAA); PTPRS_7 siRNA (SI03056284 Qiagen, target sequence: ATGGCGTGCCCGAATACCCAA).

Scrambled siRNAs from Qiagen (SI03650325) and Origene (SR30004) were used as controls. Transfections were performed at 20–30% cell confluency using the RNAiMAX Lipofectamine (Life Tech) according to the provided protocol using 30 nM of siRNA.

### Plasmid Transfection

The *PTPRS* expression vector pRK-PTPRS was kindly provided by Dr. Jeff MacKeigan (Laboratory of Systems Biology, Van Andel Research Institute, Grand Rapids, MI) (23). Cells were grown to ~50% confluency and were then treated with Lipofectamine 3000 (Cat.No.11668-019 Thermo Fisher). The *PTPRS* expression vectors containing various site and deletion mutations were also customarily ordered from GeneCopoeia.

### CRISPR knockout of PTPRS

The CRISPR kit for *PTPRS* was purchased from Origene (Cat.No.KN211163) and used according to the product protocol. Cells were transfected using Lipofectamine3000. The gRNA sequence KN211163G1, *PTPRS* gRNA vector 1 in pCas-Guide vector, (target sequence: CTTGTGGTCCTGCTCGTTGG) proved the most effective at knocking out (KO) *PTPRS* expression and was thus used to create the HCT116, HT29 and SW620 PTPRS KO cell lines. CRISPR cells were then grown for 7 passages and selected using puromycin (Life Technologies). Numerous colonies were isolated and tested for absence of *PTPRS* via Western blot and mRNA analysis.

### EGF Stimulation

*PTPRS* CRISPR KO and control cells of HCT116 parental and HCT116-*KRAS* (−/+) cell lines were plated with approximately 300,000 cells in 6-well plates with serum free RPMI. Cells were starved for 24 hours then treated with 125 ng of EGF (Cat.No.PHG0313 Thermo Fisher) in 2.5 mL serum free media (50 ng/ml). Cells were harvested at the 5, 15, and 30 minutes times points after EGF stimulation; an untreated 0 time point was used as a control.

### MEK Inhibitor Treatment

HCT116 *PTPRS* KO and control cells were plated in 6-well plates. After 24 hours the cells were treated with 5 μM of MEK inhibitors PD98509 (Cat.No.P215 Sigma). Cells treated for 24 hours were then either harvested for western blot analysis or used for immunofluorescent staining.

### Immunostaining

Immunostained slides were analyzed with a Leica DMi8. The Cherry C-terminal tagged *PTPRS* was obtained from GeneCopoeia. Duo Link (Sigma DU092008) assays were performed per manufacturer’s instructions using Mouse Ab DU092004

### Statistical Analysis

We previously analyzed 468 stages I-IV colorectal tumors with (affymetrix) global gene expression analysis data from the surgical specimen and targeted gene sequencing of 1321 cancer-related genes^5,8^. Here we further used this well-curated clinico-genomics/expression database of CRC patient samples to carry out mutation ranking analysis using SAS 9.4 (Cary, NC). We first stratified the 468 CRCs by an 18-gene RAS pathway gene expression signature score^16^. The arithmetic mean expression of the 18 signature genes of a tumor sample is designated as its 18-gene RAS pathway score. A mutated gene list was constructed by ranking the RAS signature scores of tumors with and without a mutation in the given gene (out of 1321) using the p-value coming from one-sided Wilcoxon rank sum test with normal scores, where the mutated tumors give rise to higher RAS signature scores. For cell line studies, experiments were done in triplicates, and mean and standard deviation were calculated as indicated. Two-tailed, paired *t* test was used to determine the statistical significance of comparison as needed.

### Availability of materials and data

The materials and datasets generated during and/or analyzed during the current study are available from the corresponding author on reasonable request.

## Electronic supplementary material

Supplementary Table and Figures
