# Supplementary material for: PTPRS Regulates Colorectal Cancer RAS Pathway Activity by Inactivating Erk and Preventing Its Nuclear Translocation
Source: Sci Rep. 2018 Jun 18;8:9296. doi: 10.1038/s41598-018-27584-x (PMC6006154; doi:10.1038/s41598-018-27584-x)
Supplement: Supplementary file 1 — Supplementary Table and Figures [file 41598_2018_27584_MOESM1_ESM.docx]

**PTPRS REGULATES COLORECTAL CANCER RAS PATHWAY ACTIVITY BY INACTIVATING ERK AND PREVENTING ITS NUCLEAR TRANSLOCATION**

Thomas B. Davis^1#^, Mingli Yang^1#^, Michael J. Schell^2^, Heiman Wang^1^, Le Ma^1^, W. Jack Pledger^1,3^, Timothy J. Yeatman^1^*

^1^Gibbs Cancer Center & Research Institute, 380 Serpentine Drive, Spartanburg, SC 29303 (USA);

^2^Department of Biostatistics and Bioinformatics, Moffitt Cancer Center & Research Institute, 12902 Magnolia Drive, Tampa, FL 33612 (USA);

^3^Department of Molecular Medicine, VCOM, 350 Howard Street, Spartanburg, SC 29303 (USA)

^#^These authors contributed equally to this work.

*Corresponding author: Timothy J. Yeatman, MD, Gibbs Cancer Center & Research Institute, 380 Serpentine Drive, Spartanburg, SC 29303, Tel: +1 864-560-1052, Fax: +1 864-560-1196, email: yeatman@gibbscc.org

Supplementary Table 1. **RAS pathway signature score-associated genes among 209 RAS/RAF wild-type CRC tumors**

**Gene p-value* N Pct**

*Genes with 5+% frequency*

ADAMTSL3 .00002 19 9.1

ITGB4 .00002 12 5.7

APC2 .00058 11 5.3

GNAS .00120 14 6.7

PTPRS .00181 22 10.5

ATG2B .00428 15 7.2

*Rarer, but highly associated genes (2.5-4.9%)*

TGFBR2 <.00001 7 3.3

SLC2A4 .00001 6 2.9

INSRR .00084 7 3.3

NLRP3 .00124 7 3.3

MAP3K9 .00161 6 2.9

MAPT .00191 7 3.3

MN1 .00252 6 2.9

MCM3AP .00294 8 3.8

PTK2B .00420 8 3.8

* *p*-value from normal scores test for comparing the RAS pathway signature scores of mutated and wild-type tumors for the given gene.


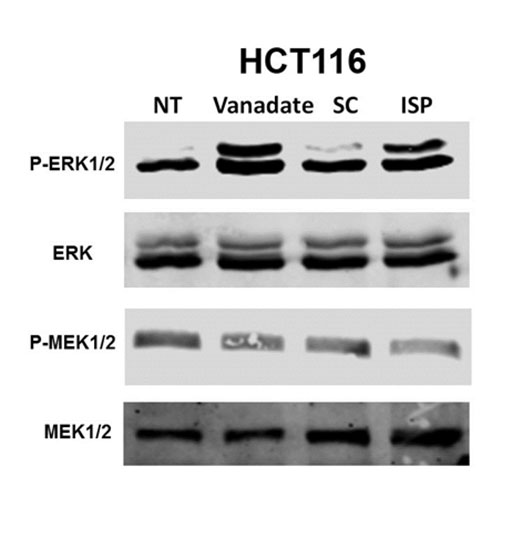


Supplementary Fig. 1. **Inhibition of PTPRS increased the phosphorylation of ERK but not MEK.**

HCT116 cells were treated with 10 μM ISP (Intracellular Sigma Peptide, the PTPRS inhibitor), Scrambled peptide (SC), no treatment (NT) for 24 hours. HCT116 cells were also treated with 10 μM vanadate, a pan inhibitor of PTPs, as a control for inhibition of PTPRS’s phosphatase activities.


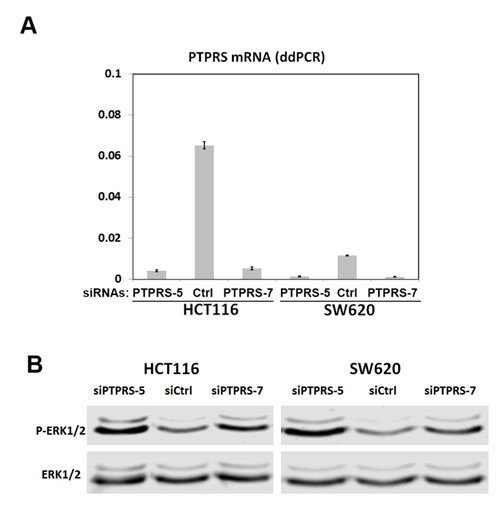


Supplementary Fig 2. **siRNA knock down of PTPRS increased the phosphorylation of ERK**.

siRNA knockdown of PTPRS in HCT116 and SW620 cells for 48 hours (Ctrl – a scrambled control siRNA; PTPRS-5 and PTPRS-7 – two independent PTPRS-specific siRNAs as described in Methods) . (**a**) The ddPCR measurement of PTPRS mRNA expression that was normalized by the expression of the reference gene B2M; (**b**) Western blot analysis of pERK1/2 and ERK1/2.


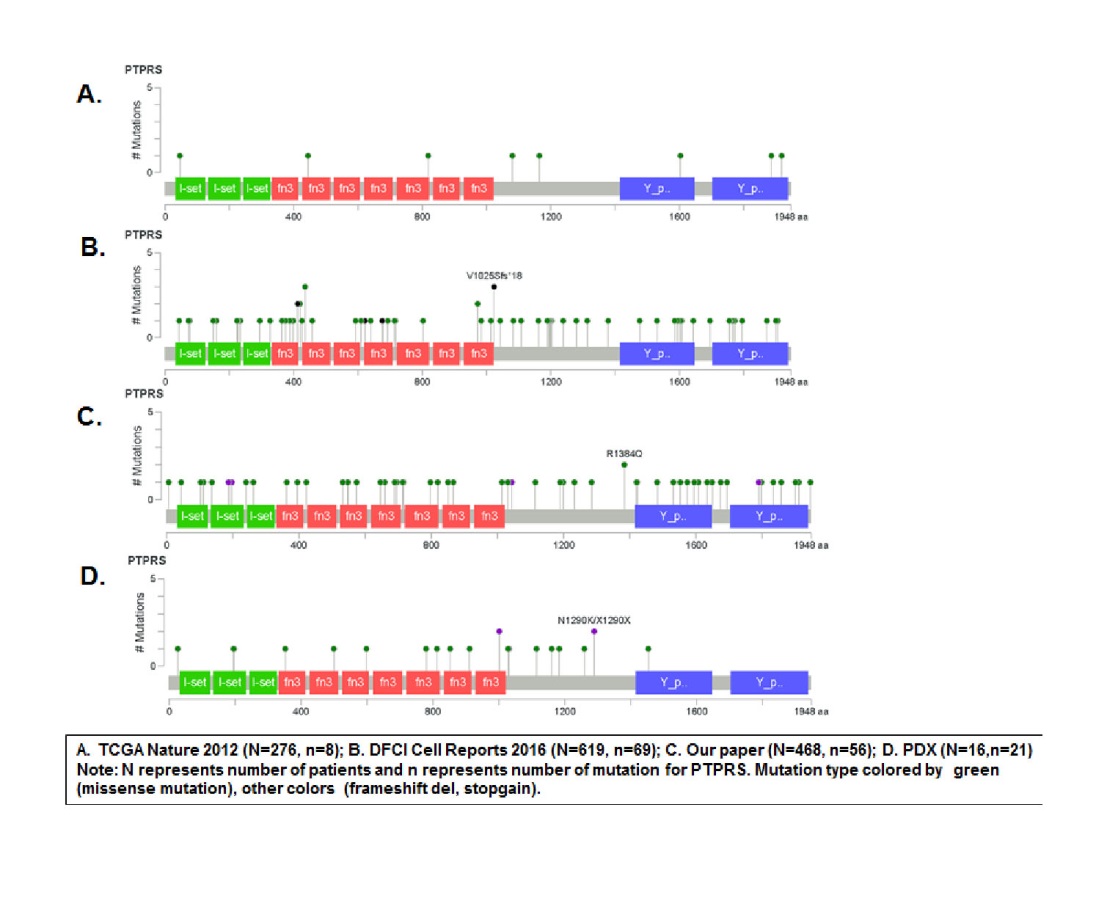


Supplementary Fig 3. **PTPRS mutation data from multiple sequencing data sets**

PTPRS is seen to be mutated in 10% of cells with mutations spanning its entire sequence as shown for the 468 CRC tumor data base and the Dana Farber CRC database.


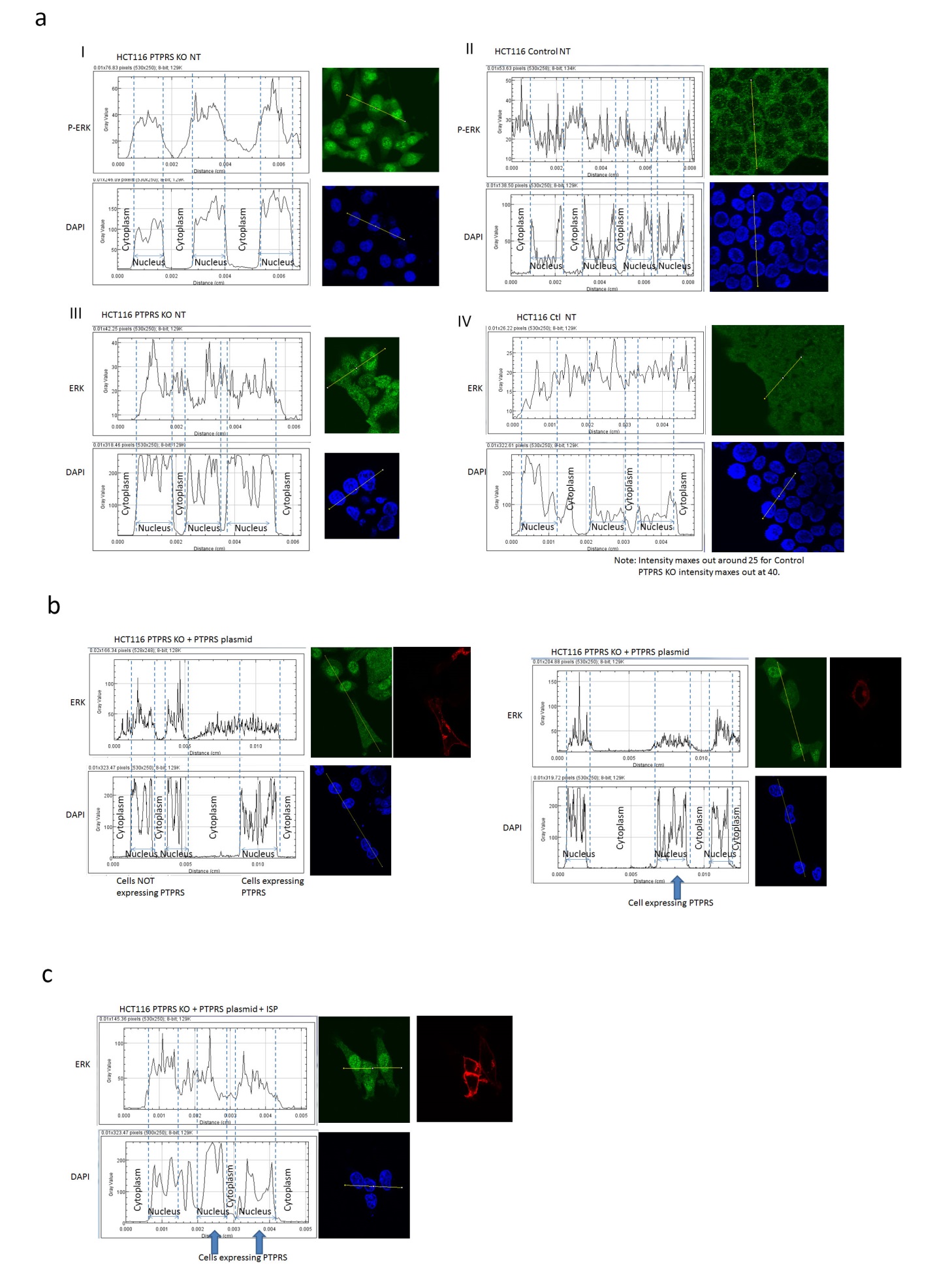
Supplementary Fig 4. **Linear profile of fluorescence intensity for immunofluorescent images of figure 8.**

A) Fluorescent images of HCT116 PTPRS KO and control cells tagged for P-ERK, ERK, and DAPI from figure 8A. Image-J program was used to analyze the fluorescent intensity of both P-ERK or ERK signal and the corresponding DAPI stain. The raw read outs in gray values are labeled with nucleus and cytoplasm in the areas of the graph that correspond to that part of the cell.

I) HCT116 PTPRS KO cells labeled with P-ERK. The signal for P-ERK gives two significantly bright peaks reaching up to 60 units in the second and third nucleus sections. The Cytoplasm sections seem to have lower levels all below 20.

II) HCT116 Control cells labeled with P-ERK. The cytoplasmic regions actually seem to show some significant spikes, while the nuclei regions seem to have dips in P-ERK.

III) HCT116 PTPRS KO cells labeled for ERK. The first and second nuclear regions show significant spikes up to 40 units. The cytoplasmic areas surround these areas seem to show a drop off.

IV) HCT116 Control cells labeled with ERK. Here we see the general read out of ERK is more constant. The scale of this read out maxes out at 25, and the KO maxed out at 40. So we see the signal is more even across the control cells with no major spikes in with the cytoplasm or nucleus.

B) Linear profile of fluorescence intensity for KM12L4A PTPRS KO transfected with PTPRS tagged with red fluorescent protein and strained for ERK (Figure 8d). Cells over expressing PTPRS (Red) show a lack of ERK (Green) in the nucleus (DAPI blue). This is confirmed by these linear profiles. In the left figure the first two nuclei show a high level of signal reaching up to 100. Conversely, the cell expressing PTPRS shows an ERK signal of only 50. The right figure shows a similar pattern.

C) HCT116 PTPRS KO cells transfected with PTPRS-RFP tagged were treated with ISP to inhibit PTPRS (figure 8f). This inhibition of PTPRS returns the ERK signal to the nucleus that is not need in figure B. This is further confirmed with the linear profile of fluorescence intensity. This readout shows peeks in ERK signal on every nuclei even those expression PTPRS.


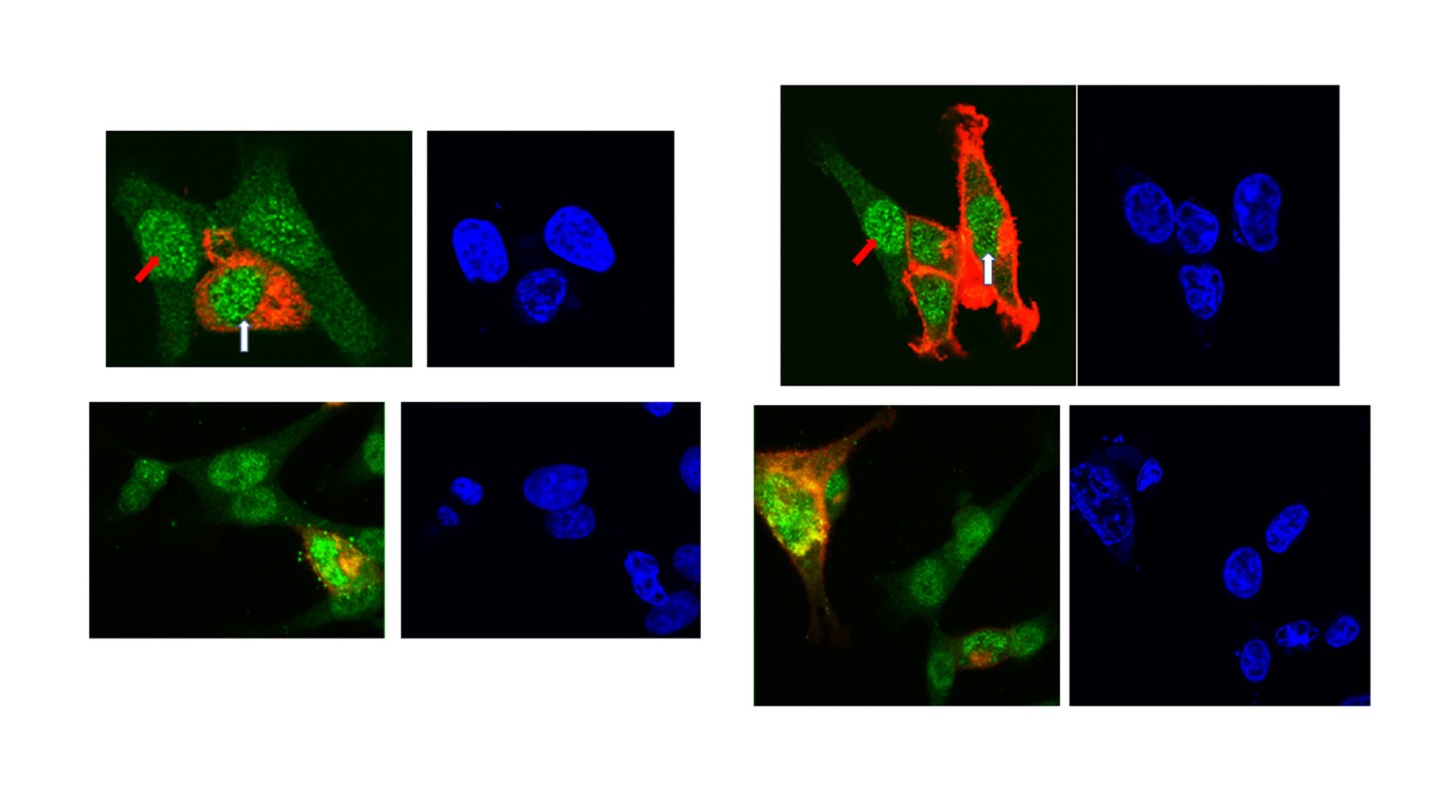
Supplementary Fig 5. **DAPI stains for figure 8f**

The DAPI stains for Figure 8f show that the intense ERK signal is coming from nucleus as expected.

**Supplementary Fig. 6**

**Full Length Blots for Figures
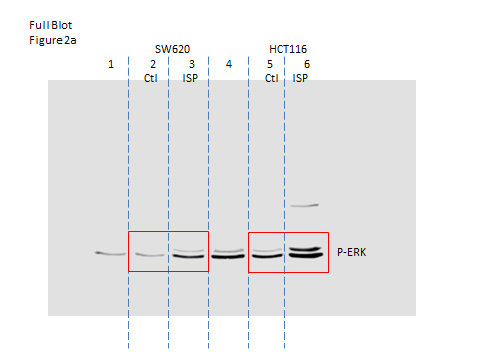

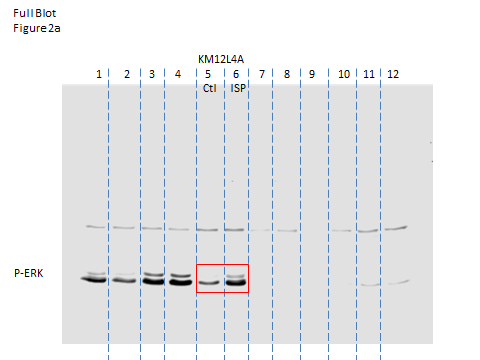

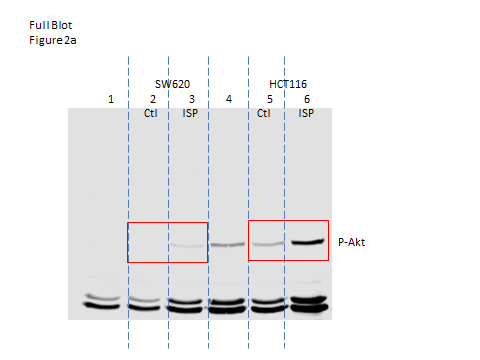

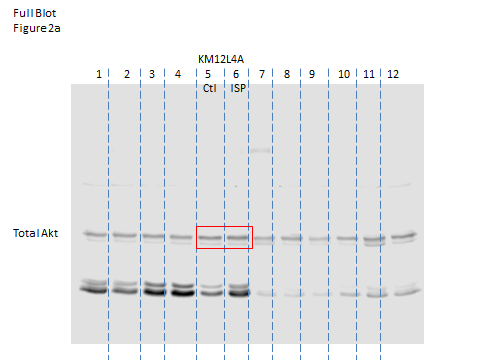

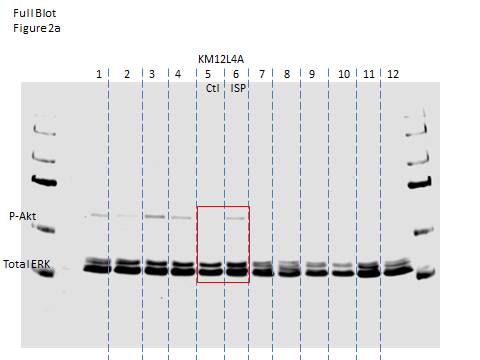

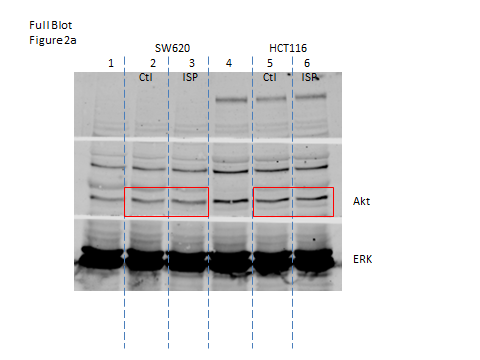

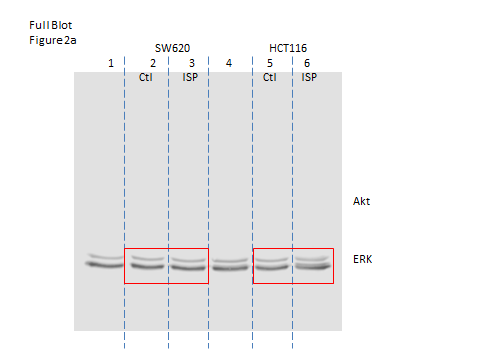

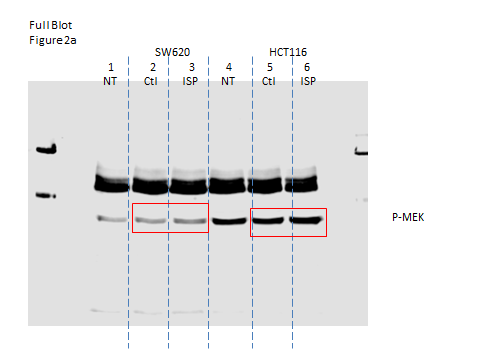

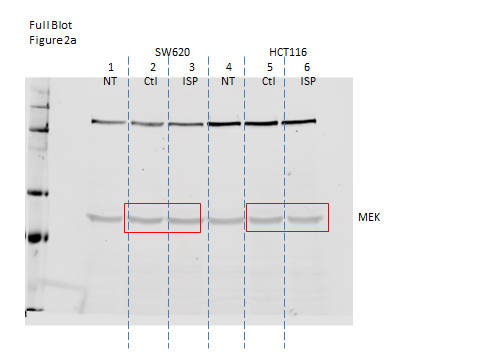

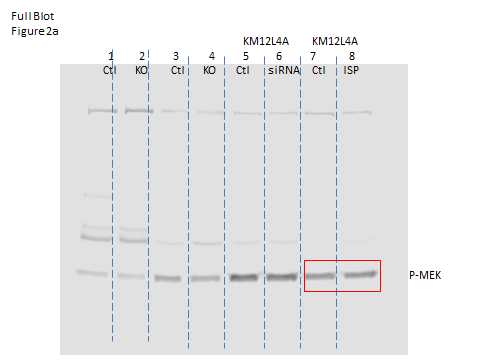

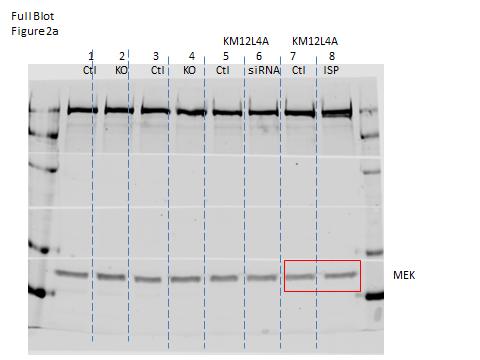

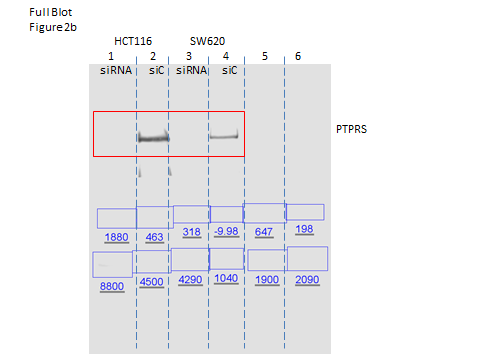

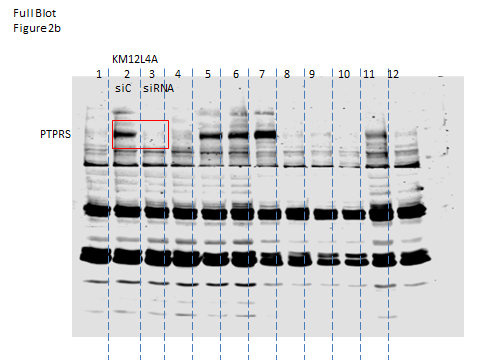

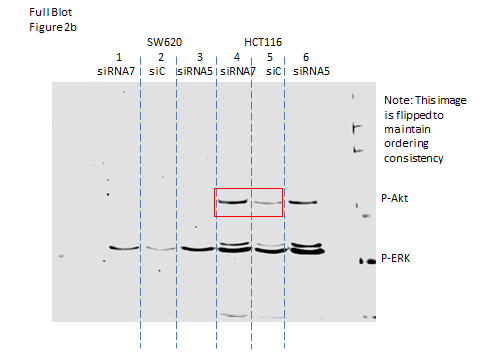

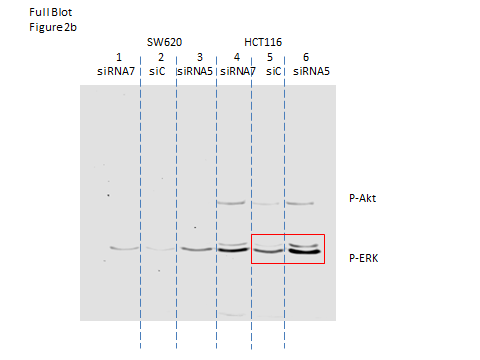

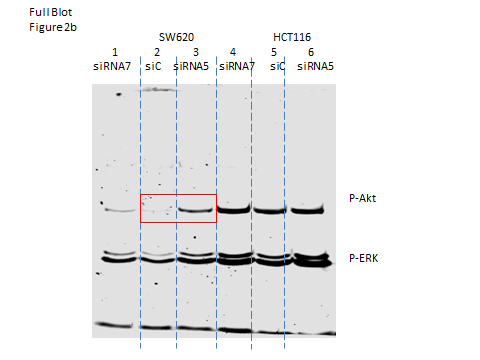

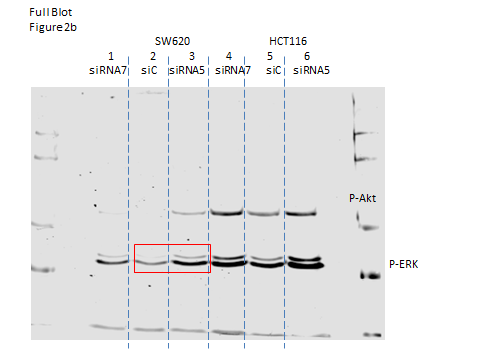

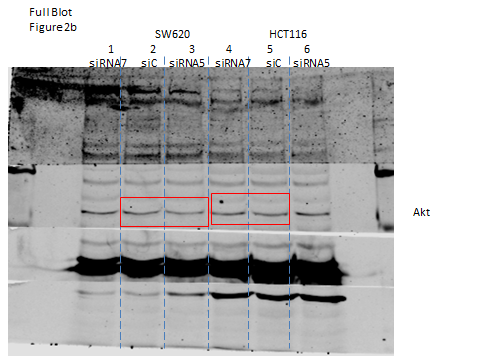

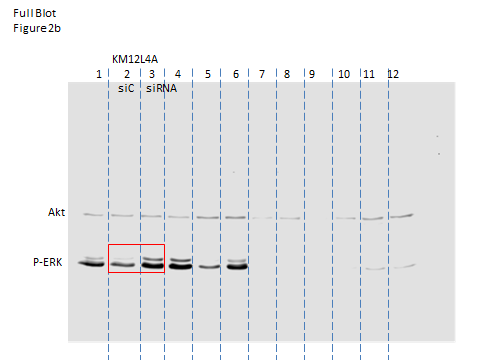

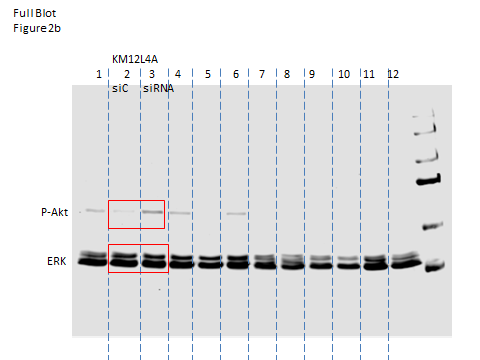

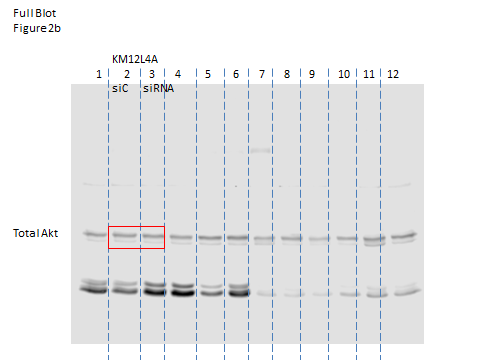

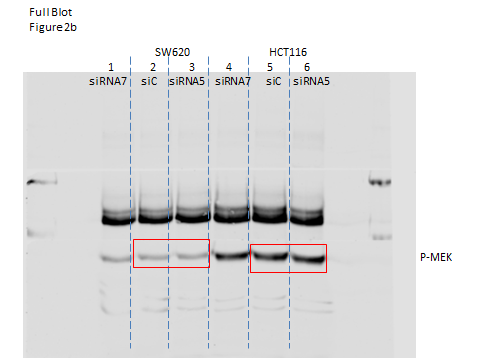

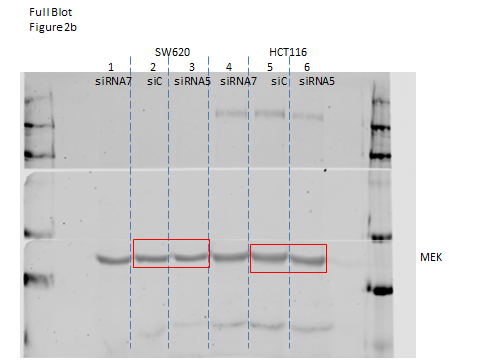

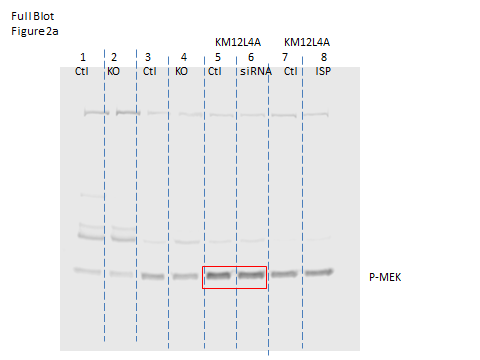

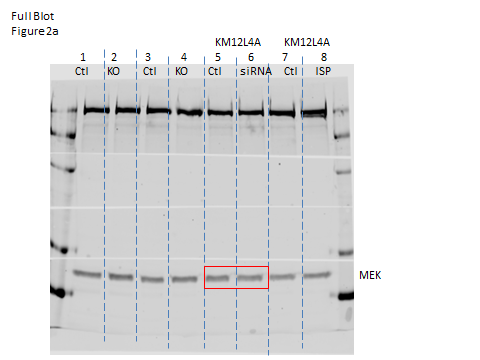

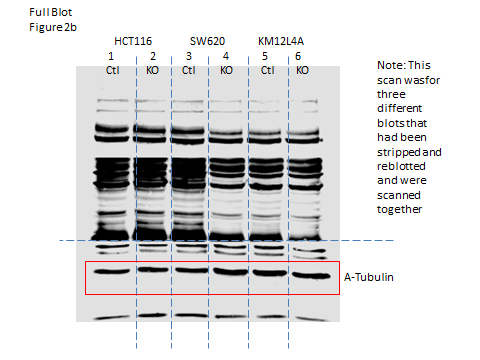

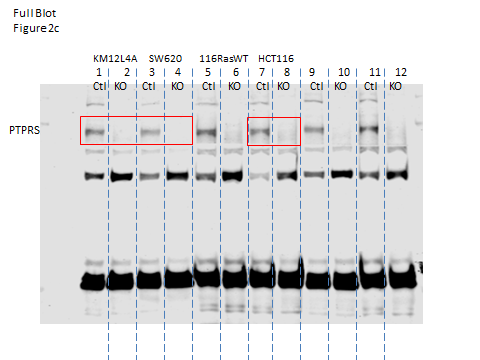

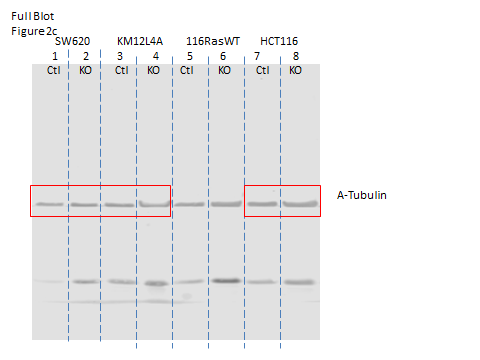

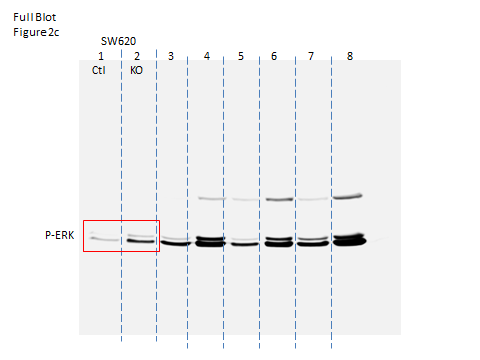

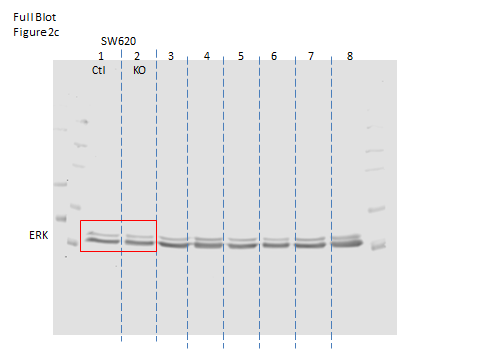

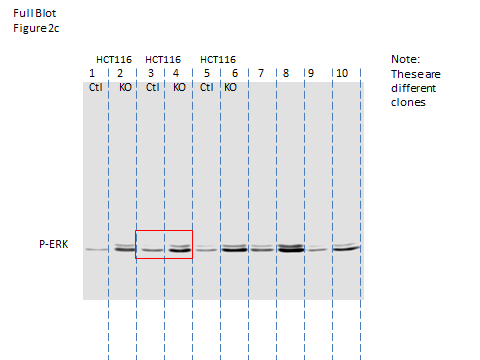

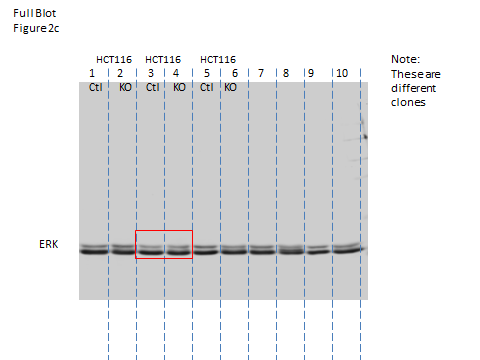

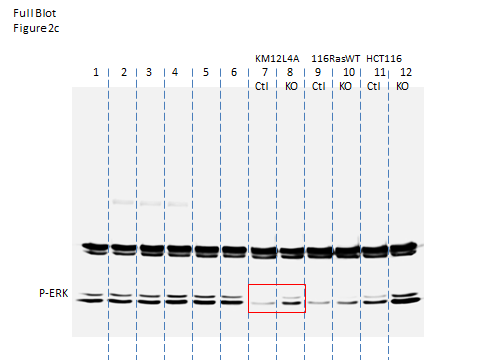

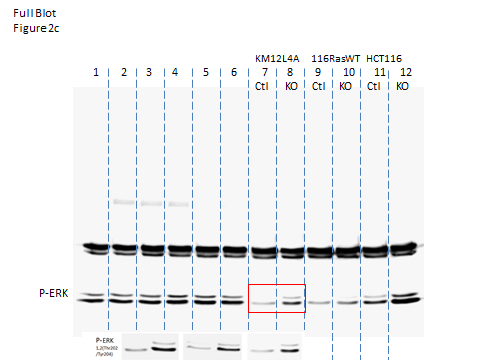

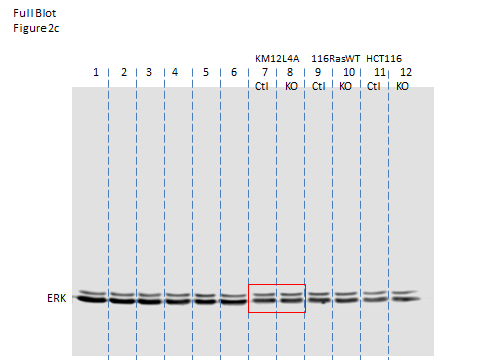

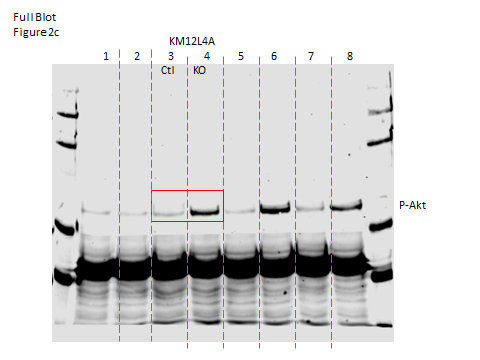

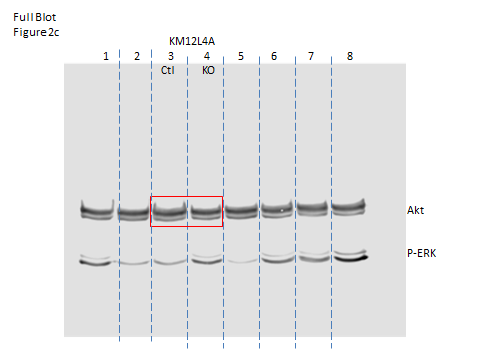

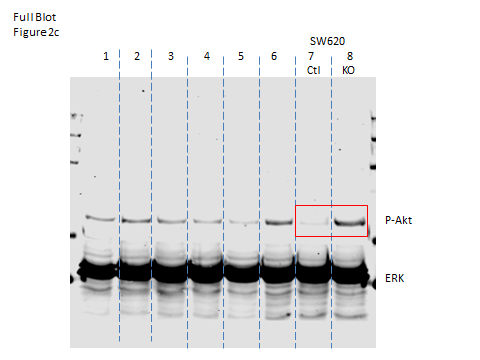

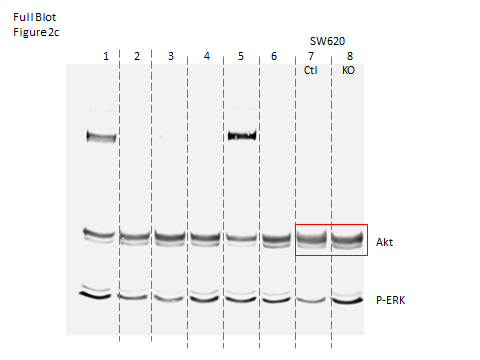

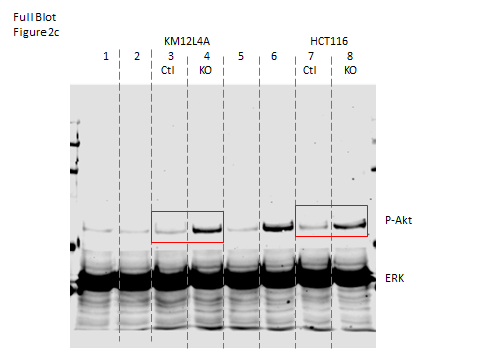

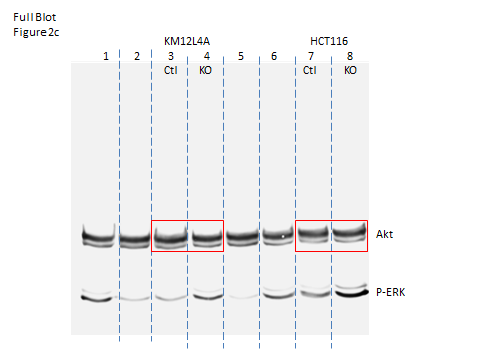

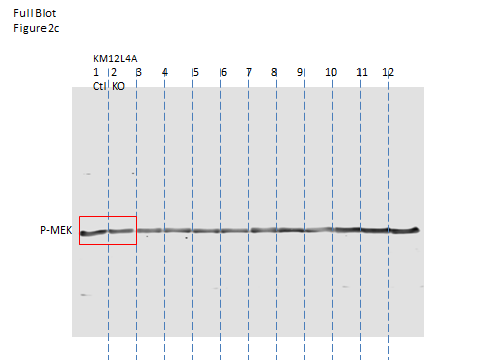

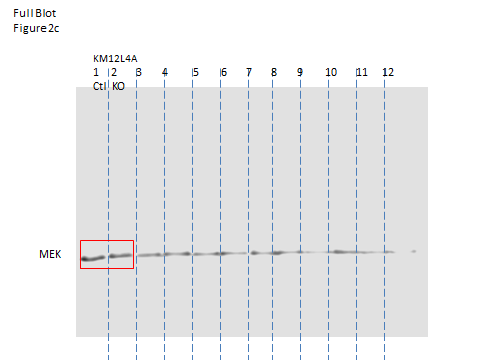

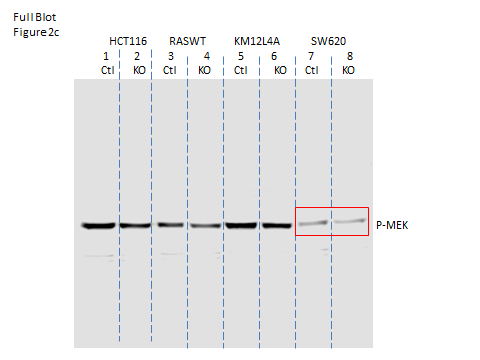

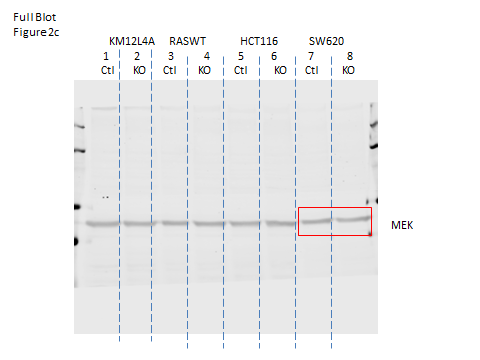

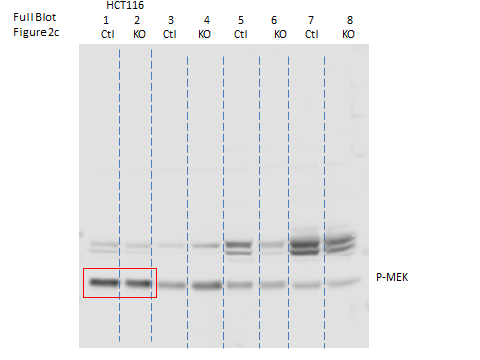

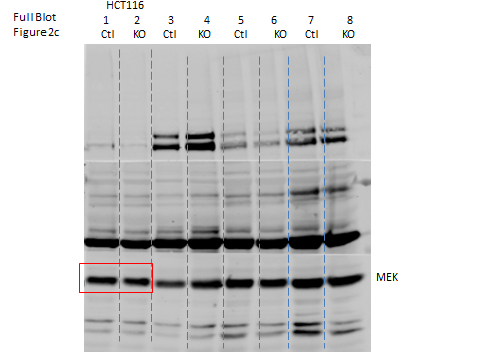

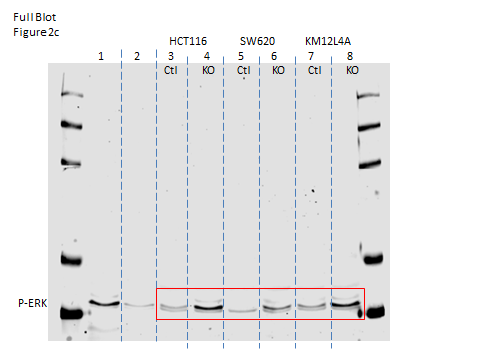

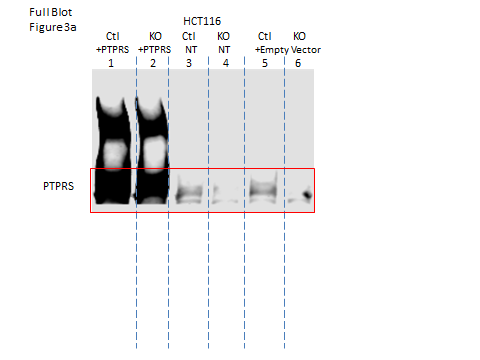

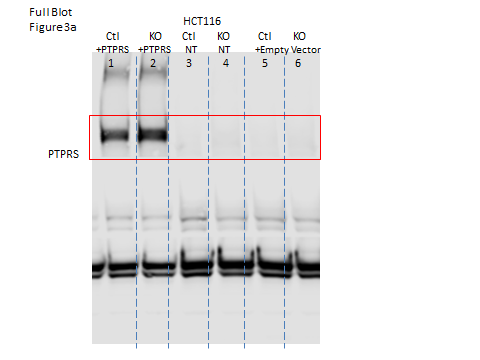

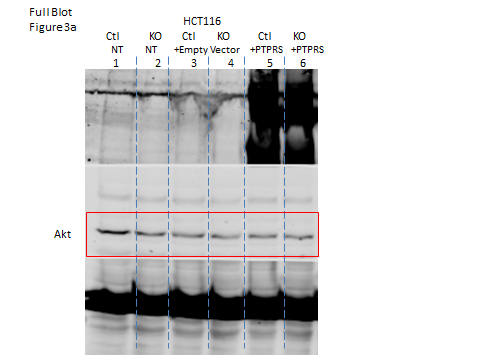

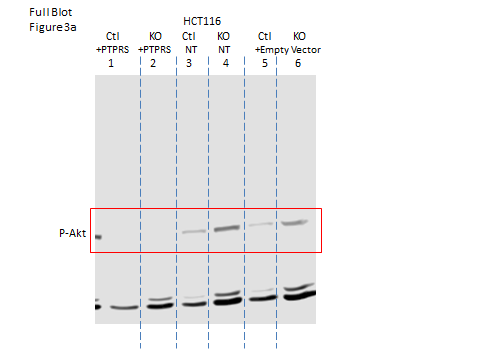

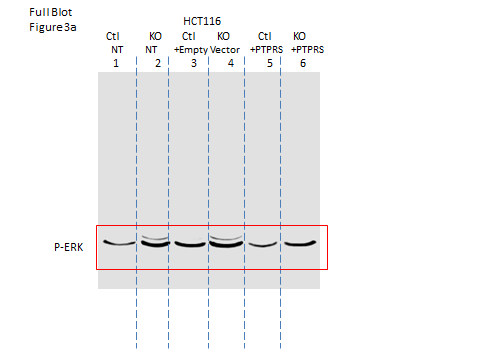

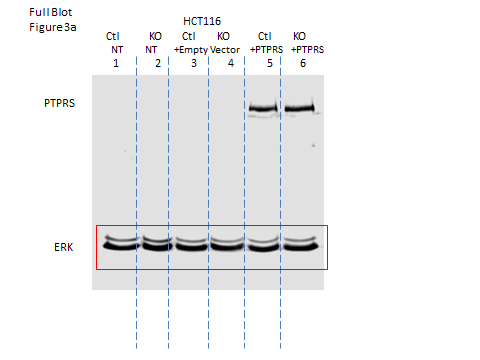

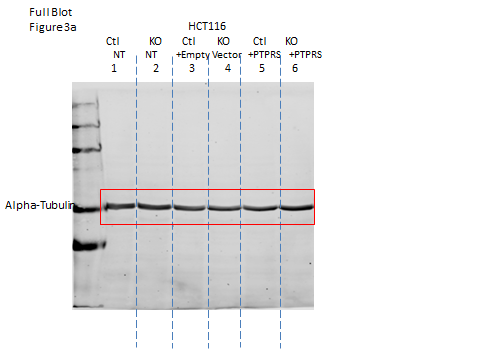

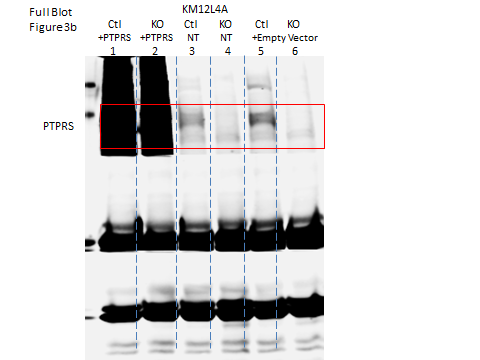

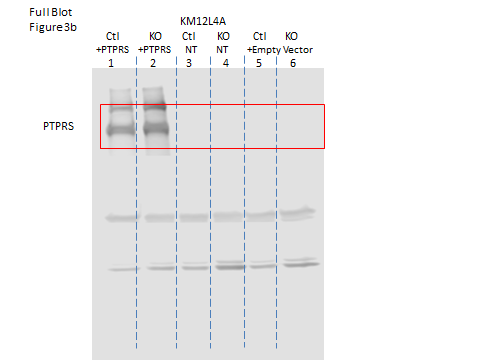

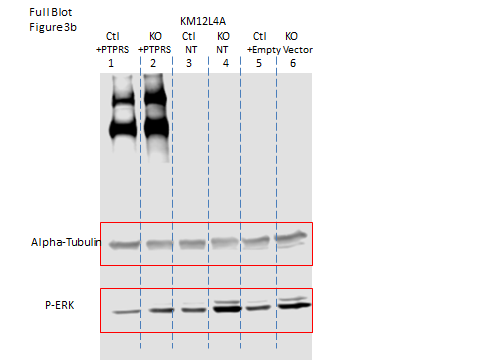

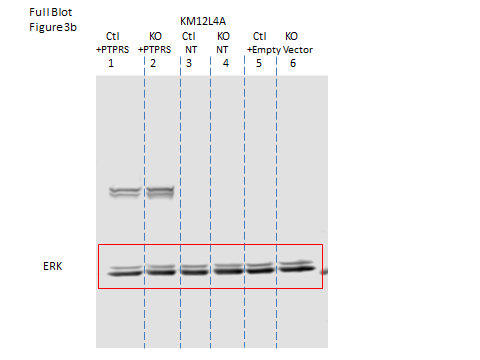

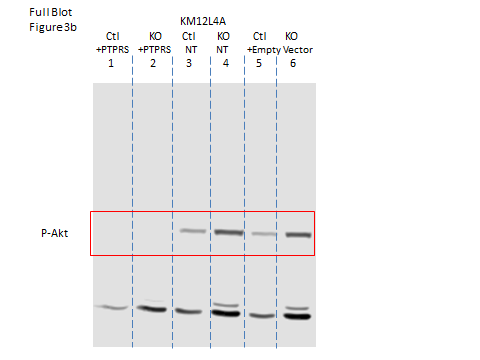

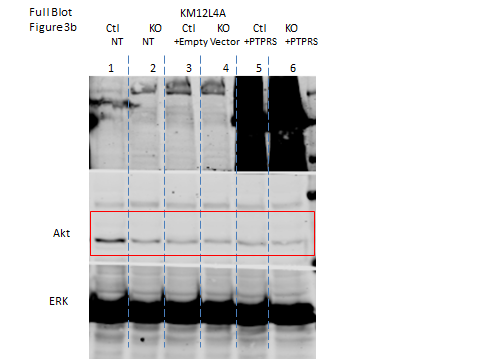

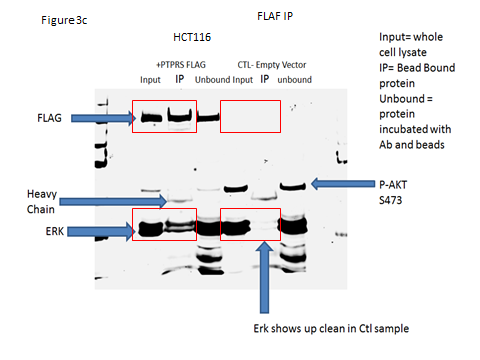

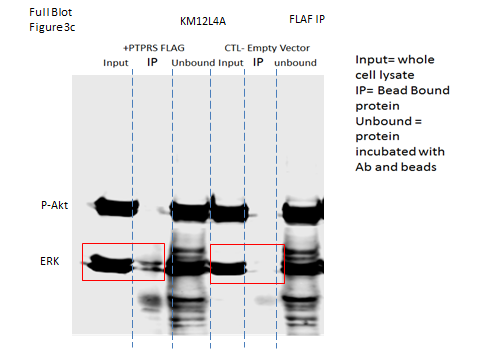

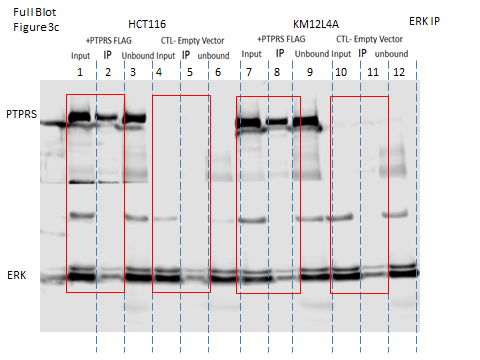

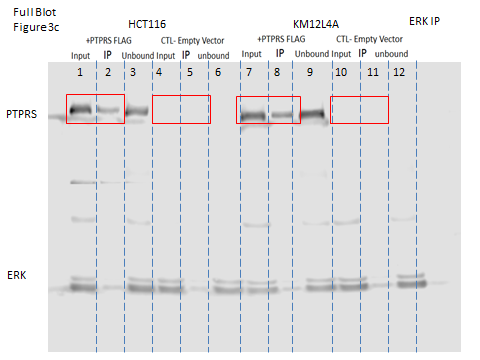

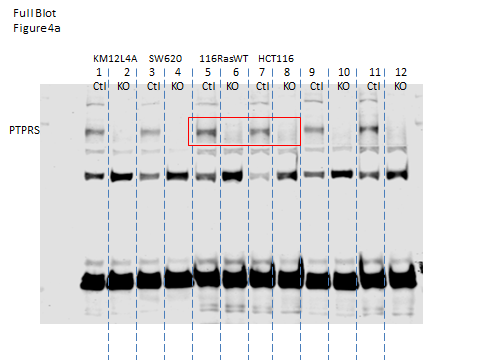

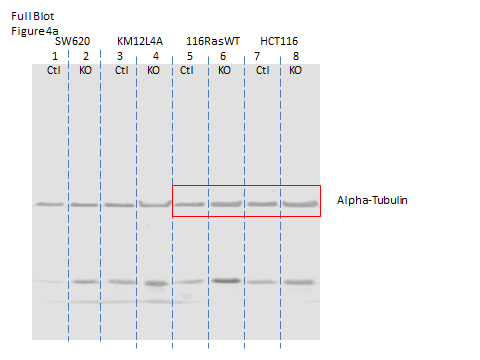

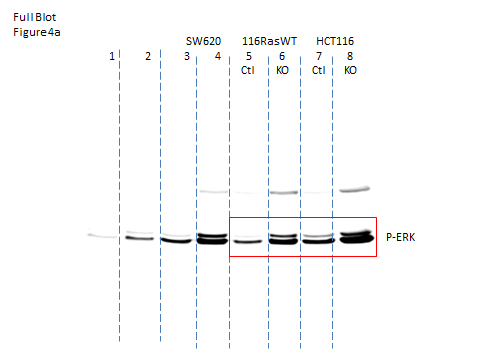

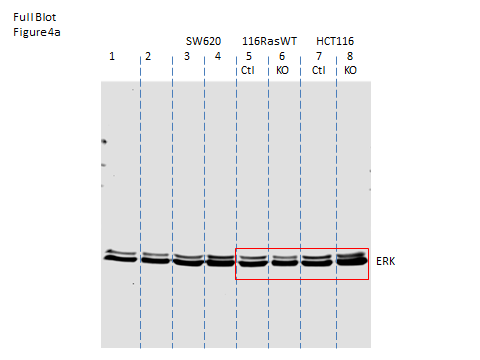

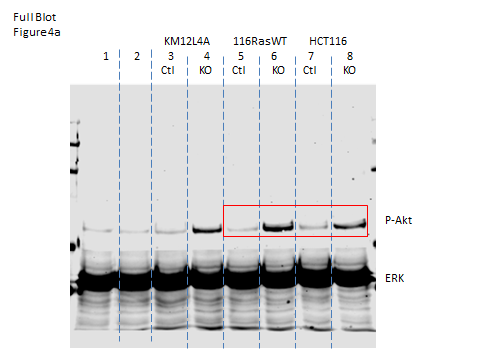

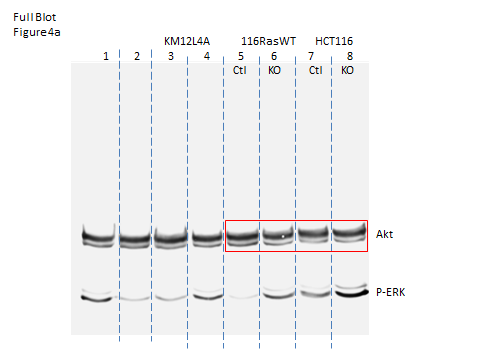

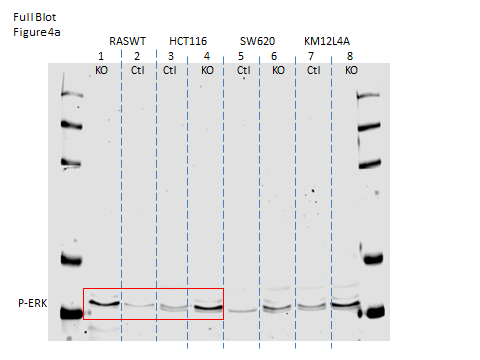

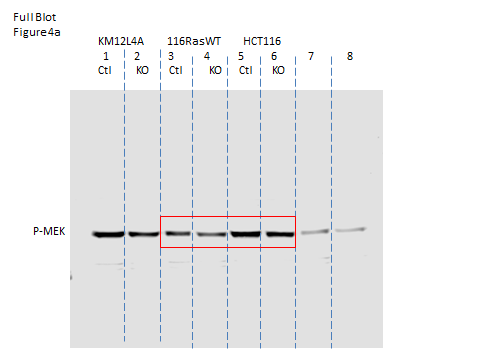

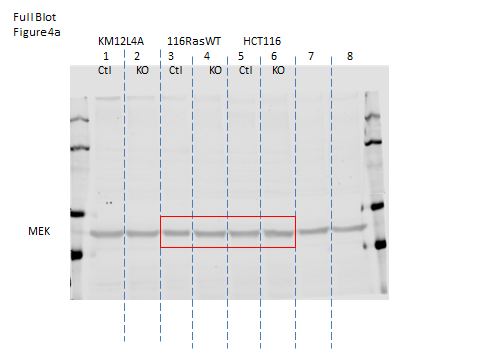

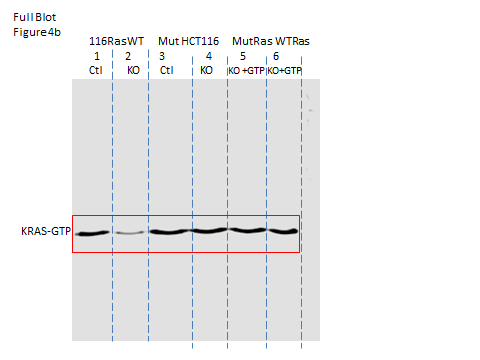

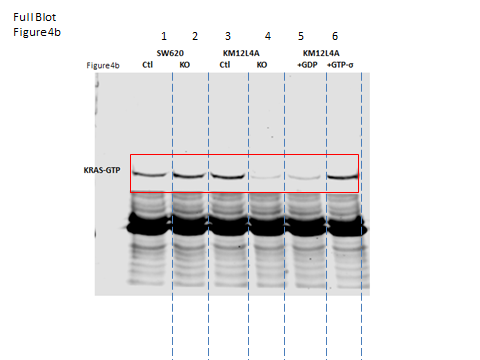

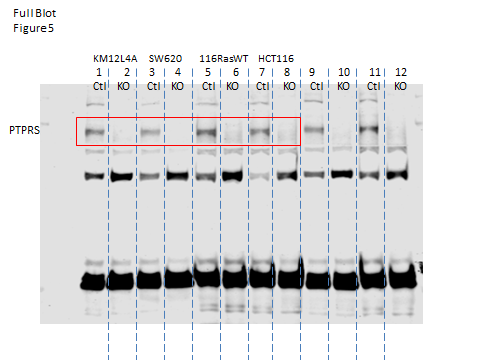

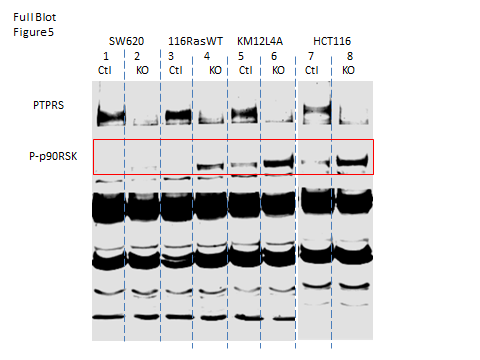

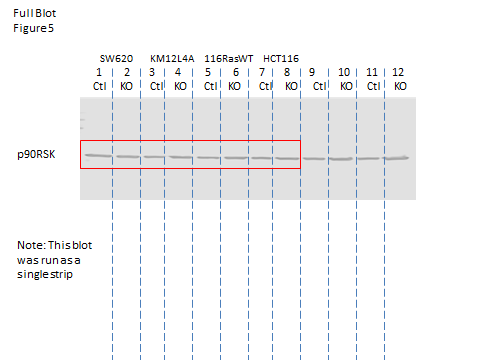

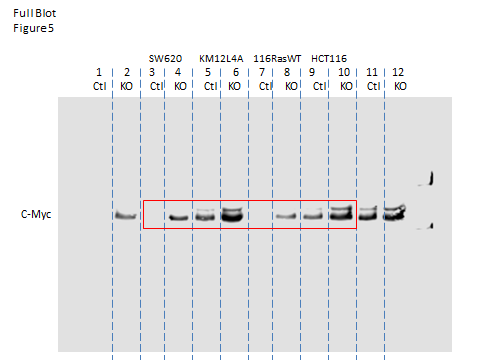

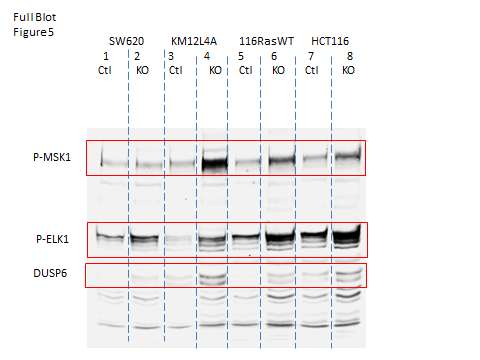

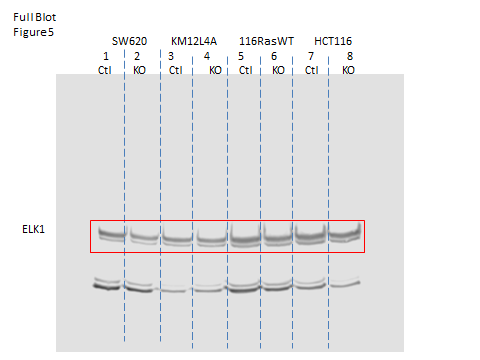

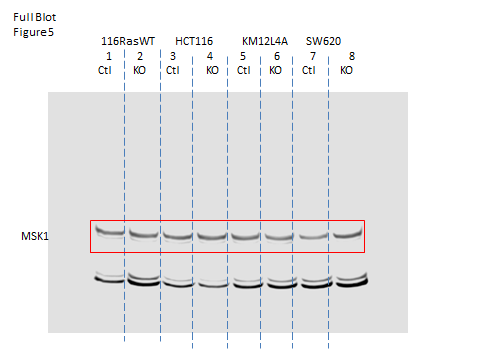

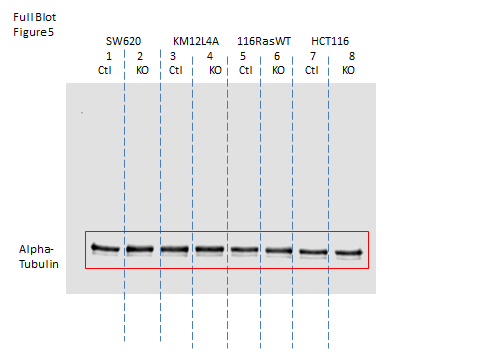

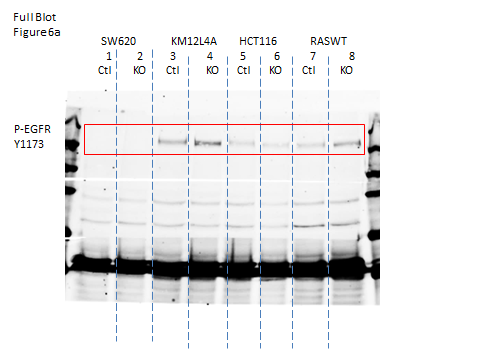

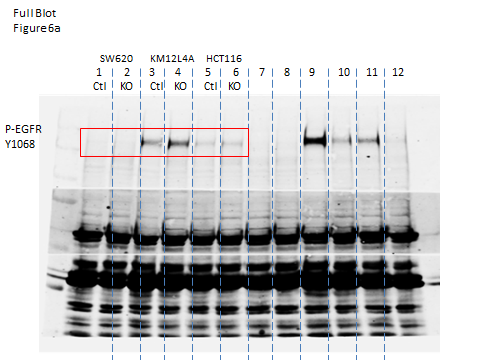

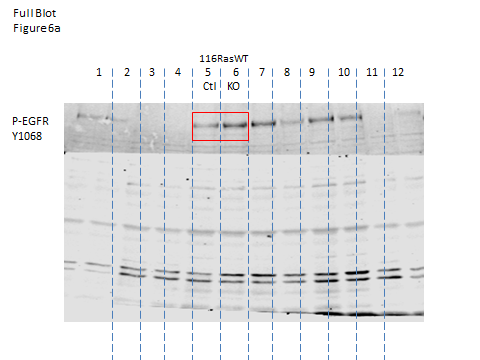

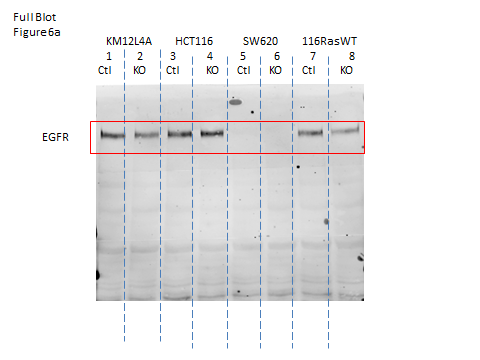

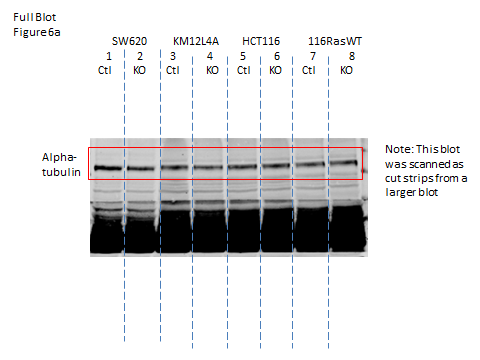

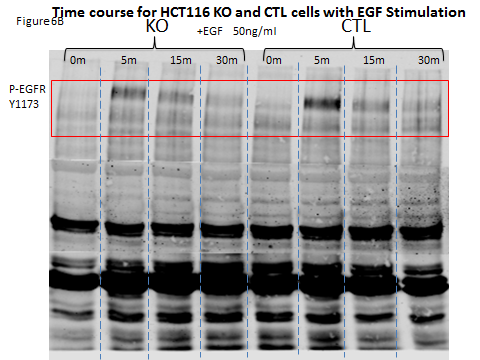

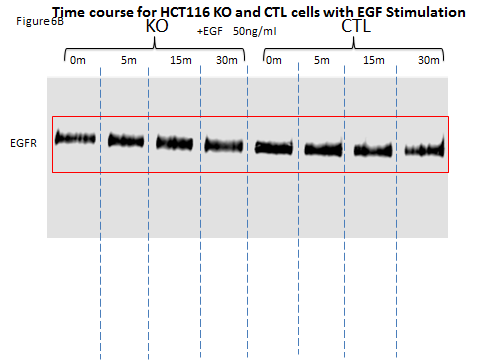

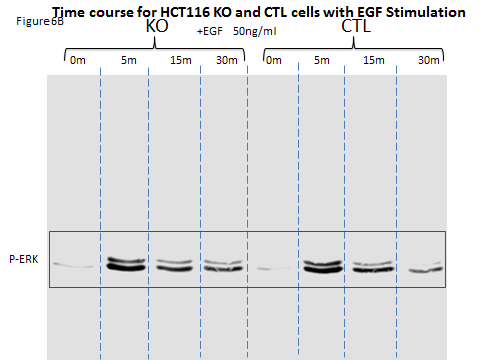

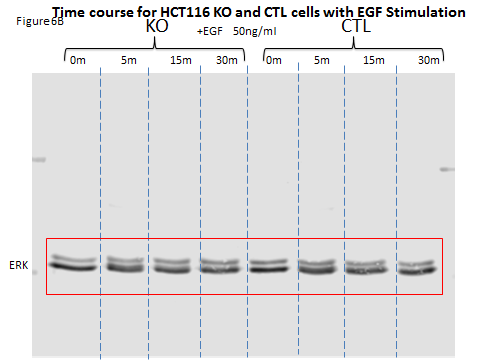

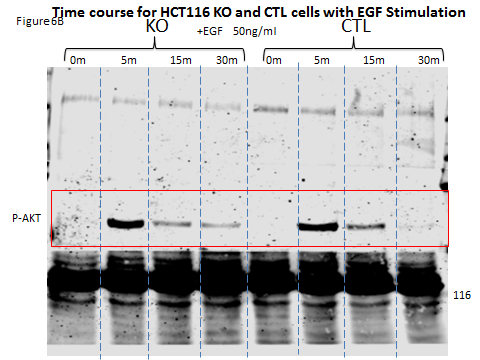

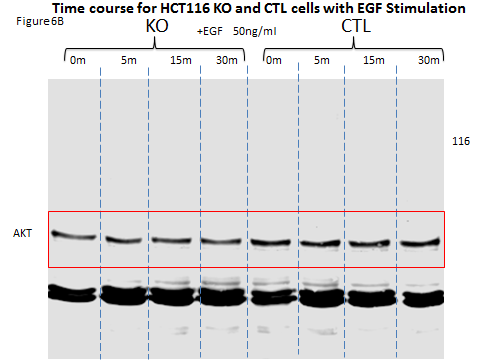
**
